# Supplementary material for: A bony-crested Jurassic dinosaur with evidence of iridescent plumage highlights complexity in early paravian evolution
Source: Nat Commun. 2018 Jan 15;9:217. doi: 10.1038/s41467-017-02515-y (PMC5768872; doi:10.1038/s41467-017-02515-y)
Supplement: Supplementary file 1 — Supplementary Information [file 41467_2017_2515_MOESM1_ESM.pdf]

Supplementary Note 1: Provenance and authenticity of PMoL-B00175

PMoL-B00175 was collected by Mr. Yang Jun, a local farmer from Qinglong County, Hebei Province, China, and acquired by the Paleontological Museum of Liaoning in February, 2014. Mr. Yang Jun reported that he collected the specimen from a quarry near Nanshimenzi Village in Gangou Township, Qinglong County, Hebei Province where lacustrine beds of the early Late Jurassic Tiaojishan Formation are exposed [1].

Our research team has organized several expeditions to localities near Nanshimenzi and Nanshimencun villages in Gangou Township (Supplementary Fig. 1) and near Bawanggou Village in Mutoudengzi Township, Qinglong County, Hebei Province. These localities outcrop the lower Upper Jurassic Tiaojishan Formation and have yielded numerous fossils of the Yanliao Biota [2]. Important vertebrate fossils from these localities include the salamander *Chunerpeton tianyiensis*[3] and *Qinglongtriton gangouensis*[4], the 'rhamphorhynchoid' (i.e. non-pterodactyloid) pterosaurs *Changchengopterus pani*[5], *Qinglongopterus guoi*[6], and *Dendrorhynchoides mutoudengensis*[7], the theropod *Yi qi*[8], and the haramiyidan mammal *Arboroharamiya jenkinsi*[9], among others.

The quarry producing PMoL-B00175 is near Nanshimenzi Village (Supplementary Fig. 1), and it is also called Zhuanshan Locality or Gangou Locality in some literatures [10]. In order to confirm the provenance information from Mr. Yang, we carried out an excavation at this locality in the summer of 2016 (Supplementary Fig. 1b), which resulted in multiple fossils of plants, invertebrates and salamanders (Supplementary Fig. 2). These fossils are common elements for the Yanliao Biota and provide strong evidence supporting the Jurassic age of the fossil-bearing beds.

More importantly, our geological investigations reveal that the fossil-bearing layers at this locality are nearly identical to the host matrix of PMoL-B00175 (Supplementary Fig. 3), both displaying a unique combination of features not known in most other localities in western Liaoning, southeastern Inner Mongolia, and northern Hebei: dark grey shales, a proportionally high content of fine sand, and rich in dark plant fragments. These observations

also strongly support the provenance of PMoL-B00175 from the Nanshimen Locality.

PMoL-B00175 is an articulated skeleton with associated fossilize soft tissues, preserved in slab and counter slab (Supplementary Fig. 3). Unlike most specimens collected by local farmers, which are often visibly in many small pieces that cemented together and mounted on a supporting larger block, neither the slab nor counter slab show shims, added matrix or cement. Instead, the slab comprises six separate pieces, which can be assembled perfectly into one continuous, thick block matching the counter slab. All skeletal and soft tissue structures crossing fracture lines on the slab and counter slab do so smoothly and naturally. Finally, all skeletal elements, soft tissues and the host matrix as well from the slab completely mirror the corresponding structures preserved on the counter slab (Supplementary Fig. 3).

Both the slab and counter slab have only been prepared by two professional technicians (Xiaoqing Ding and Matthew Brown) under the supervision of the senior authors (XX, DYH and JC). The preparations exposed a substantial portion of the specimen, including both skeletal elements and soft tissues. Taken together, our data strongly support the authenticity of the specimen and support the validity of all morphological data recovered from the specimen.

Supplementary Note 2: Geological ages of Yanliao theropods

The Middle-Late Jurassic Yanliao Biota has produced fossil remains of several feathered dinosaurs over last 10 years [2, 3, 8, 10, 11, 12, 13, 14, 15, 16, 17]. These discoveries are significant in understanding early paravian evolution, and particularly the origins of birds and related issues [18]. However, the geological ages of the fossil-bearing beds and their correlations have been somewhat controversial [2, 3].

Important vertebrate fossils of the Yanliao Biota were first recovered from the Daohugou beds of Inner Mongolia [19, 20], but the correlation of the beds to other well-known Yanliao deposits from the same geographical region are debated, and the proposed geological ages of the Daohugou beds range from the Middle Jurassic through Early Cretaceous [2, 3, 10, 21]. Recently, a consensus has been reached, and the Daohugou fossil-bearing beds are suggested to be mainly Callovian in geological age and referable to the Haifanggou/Jiulongshan Formation [2, 10]. More

recently, significant vertebrate fossils have also been recovered from the Tiaojishan Formation of several localities particularly a few localities in Linglongta of western Liaoning, which is probably Oxfordian in geological age [10, 22]. Although a consensus on the geological ages of the fossil-bearing beds in different Yanliao localities and their correlations have yet to be reached, it is now clear that the Yanliao Biota lasted from Callovian (or slightly earlier) through Oxfordian, and the biota can probably be further divided into the Callovian Daohugou fossil assemblage and the Oxfordian Linglongta fossil assemblage [10].

The Daohugou fossil assemblage covers a relatively large geographical area, including Daohugou, Ningcheng, southeastern Inner Mongolia, Reshuitang, Linyuan, western Liaoning, and Mutoudeng-Gangou, Qinglong, northern Hebei, among others. The Linglongta fossil assemblage has a relatively restricted geographical range, and so far most Linglongta theropod fossils have been recovered in Jianchang County, western Liaoning (e.g., the Daxishan Locality in Linglongta, Jianchang County, Liaoning, and the Yaolugou Locality in Yaolugou, Jianchang County, Liaoning). The Mutoudeng-Gangou Locality, which has produced the scansoriopterygid *Yi qi* fossil [8] and the new fossil described in the present paper, has been suggested to expose the Tiaojishan Formation [1], though a recent study suggests that this locality outcrops the Haifanggou/Jiulongshan Formation instead [10].

Supplementary Note 3: Relationships of *Caihong juji* to other Yanliao non-scansoriopterygid theropods

The first discovered Yanliao non-scansoriopterygid theropod is *Anchiornis huxleyi*, which was originally suggested to be a basal avialan [23]. This systematic hypothesis has been supported by several recent studies [17, 24], but a troodontid affinity [12, 25] and a deinonychosaurian affinity [13] have also been proposed. Since the description of *Anchiornis huxleyi*, several other extremely similar species have also been reported from the Tiaojishan Formation [12, 13, 16, 17, 23].

The Yanliao non-scansoriopterygid theropods either form a monophyletic group at the base of the Troodontidae [12, 25] or Deinonychosauria [13], or the Avialae [17, 23, 24], or a grade at the base of the Avialae [17]. In general, all Yanliao non-scansoriopterygid theropods are very similar in general morphology to *Archaeopteryx* [13], and even share with the latter some unique features

[13]. However, Yanliao non-scansoriopterygid theropods also share some derived features with basal deinonychosaurs or basal troodontids [13].

Caihong juji differs significantly from other Tiaojishan theropods, but meanwhile closely resembles the latter in general morphology (see the data matrix for a complete comparison between *Caihong* and other basal paravians including *Archaeopteryx* and other Yanliao non-scansoriopterygid theropods). These morphological features support the presence of an endemic theropod clade (here named as the Anchiorninae, defined as the most inclusive clade including *Anchiornis huxleyi* but not *Archaeopteryx*, *Gallus*, *Troodon*, *Dromaeosaurus*, *Unenlagia*, and *Epidexipteryx*), and this phylogenetic hypothesis has been proposed by a recently published phylogenetic analysis [26].

In order to assess the systematic position of *Caihong*, we ran an analysis on a dataset modified from a recently published study [8] with several taxa including *Caihong* added in (Supplementary Data 1). The data matrix was edited in Winclada (ver 1.00.08) and analyzed using TNT software package [27]. All multistate characters are unordered. The analysis was run using a traditional search strategy, with default settings apart from the following: maximum trees in memory 100000 and 1000 replications. The analysis produced 192 most parsimonious trees with a tree length of 1449 steps, a CI of 0.31, and a RI of 0.73 (the CI and RI were calculated separately using the WSTATS). Supplementary Fig. 18 shows the strict consensus of these 192 most parsimonious trees. We also ran bootstrap analysis on the data matrix to evaluate the robustness of the recovered clades, using TNT with all default settings except that 1000 replications were used, and bootstrap values for all the recovered clades are shown in Supplementary Fig. 19. It is notable that only a few clades have a bootstrap value greater than 50% in the present analysis, suggesting that the recovered clades are not strongly supported.This phylogenetic analysis recovers the endemic theropod clade Anchiorninae and places *Caihong* as the sister taxon to *Xiaotingia* within the Anchiorninae. Diagnostic features for the Anchiorninae include vaned feathers on forelimb symmetric, snout apering to an anterior point, quadrate strongly inclined anteroventrally, roots of dentary and maxillary teeth circular in cross-section, dentary teeth fewer than 12, dental serration large, neural spines on

posterior dorsal vertebrae in lateral view anteroposteriorly expanded distally, acromion process reduced not to contact coracoid, acromial margin of scapula laterally everted, capulocoracoid dorsal margin with pronounced notch between acromion process and coracoid, radius and ulna well separated, posterior flange on manual phalanx II-1 present, length of manual phalanx III-1 twice greater than length of IV-1, antitrochanter posterior to acetabulum absent or poorly developed, ischium curved dorsally (posterodorsally concave), ventral notch between obturator process and shaft of ischium present, pubis moderately posteriorly oriented, anterior surface of pubic shaft convex in lateral view, medial surface of proximal end of fibula flat, length of pedal phalanx II-2 greater than length of phalanx II-1, and pedal unguals III and IV strongly curved.

We further investigated the position of *Caihong* using an additional dataset with increased taxonomic sampling [26] (Supplementary Data 2). As in the first analysis, all characters are unordered. We analyzed the matrix using a new technology search strategy, and the analysis produced 22 most parsimonious trees with a tree length of 3259 steps, and these 22 trees were further analyzed using TBR branch swapping, which resulted in 100000 total MPTs. Supplementary Fig. 20 shows the strict consensus of these 100000 most parsimonious trees.

We ran the third analysis on the Supplementary Data 2, but with some multistate characters ordered following Supplementary ref. [28], and Supplementary Figs. 21, -22 show a strict consensus and a reduced strict consensus, respectively, of the 100000 most parsimonious trees resulting from this analysis. Both the second and third analyses place *Caihong* within a monophyletic Anchiorninae. However, the second analysis places *Caihong* as the sister taxon to *Xiaotingia* as the first analysis does, but the third analysis places *Caihong* within the Anchiorninae in a not-resolved polytomy with *Xiaotingia*, *Anchiornis*, *Eosinopteryx*, and *Aurornis*.

Supplementary Table 1: Selected measurements of the *Caihong juji* holotype specimen.

Skull	67.6*
Cervical series	72*
Dorsal series	87.8*
Sacrial series	31.5*
Caudal series	178*
Scapula	?/>33
Humerus	?/42.1
Ulna	?/47.2
Metacarpal II	8.6/9.3
Metacarpal III	23.2/23.7
Metacarpal IV	23.2/23.5
Manual phalanx II-1	18.2/21.8
Manual phalanx II-2	?/7.5
Manual phalanx III-1	11.6/11.8
Manual phalanx III-2	19.9/20
Manual phalanx III-3	?/10.6
Manual phalanx IV-1	5.4/5.9
Manual phalanx IV-2	5.7/5.8
Manual phalanx IV-3	13.3/13.0
Manual phalanx IV-4	7.8/7.8
Ilium	31/?
Ischium	20.5/?
Pubis	54.9/?
Femur	70.9/?
Tibia	82.8/81.6
Metatarsal I	?/5.5
Metatarsal II	47.3/47.6
Metatarsal III	49.0/?
Metatarsal IV	46.6/?
Metatarsal V	10.4/10.2
Pedal phalanx I-1	?/4.2
Pedal phalanx I-2	?/4.4
Pedal phalanx II-1	8.0*/8.7
Pedal phalanx II-2	9.6/?
Pedal phalanx II-3	10.5/?
Pedal phalanx III-1	12.2/?
Pedal phalanx III-2	8.4/8.0
Pedal phalanx III-3	8.9*/8.9
Pedal phalanx III-4	>8.6/?
Pedal phalanx IV-1	8.2/?
Pedal phalanx IV-2	7.2/?
Pedal phalanx IV-3	5.7/?

Pedal phalanx IV-4	6.4/6.2
Pedal phalanx IV-5	>5.8/8.3
Tail feathers (longest)	>112
Primaries (proximal)	>95.6 (97*)
Secondaries	>45
Feathers near chest	42
Tibial feathers (longest)	93*
Metatarsal feathers (longest)	31*

Measurements are in mm; * refers to estimated value; manual digit identifications follow the II-III-IV hypothesis.

Supplementary Table 2: List of sampled extant bird species with platelet-type melanosomes added to the core dataset (All data uploaded to DataDryad.org at 10.6084/m9.figshare.5427214 and 10.6084/m9.figshare.5427244.).

Species	Patch
Adelomyia melanogenys	gorget
Aglaiocercus kingi	gorget
Archilochus colubris	gorget
Calypte anna	gorget
Calypte costae	gorget
Chalcostigma herrani	gorget
Chrysolampis mosquitus	crown
Chrysolampis mosquitus	gorget
Coeligena bonapartei	gorget
Coeligena coeligena	gorget
Coeligena helianthea	gorget
Coeligena iris flagrans	gorget
Coeligena phalerata	gorget
Coeligena violifer	gorget
Eugenes fulgens	gorget
Eupherusa eximia	gorget
Florisuga mellivora	gorget
Heliomaster longirostris	gorget
Lamprolaima rhami	gorget
Lesbia victoriae	gorget
Phaethornis guy	gorget
Phaethornis ruber	gorget
Phaethornis yaruqui	gorget
Selasphorus sasin	gorget
Psophia crepitans	contour
Collocalia esculenta	covert
Collocalia esculenta	crown
Hemiprocne mystacea	covert
Hemiprocne mystacea	crown
Mearnsia picina	covert
Mearnsia picina	crown
Pharomachrus pavoninus	covert

Supplementary Table 3: Quadratic discriminant function analysis performance. Table shows overall results of a MANOVA used in the quadratic discriminant analysis as well as model predictive performance for six separate training datasets (see below). Prediction values in the self-test and in cross validation are similar to those in previous analyses [29, 30] and on par with performance in similar studies. In datasets 4 and 5, platelet-shaped microbodies were inferred from SEM images (i.e. whether overlapping moulds or stacked/flattened 3D solid bodies were observed; see Fig. 5a-c). The overall performance of the discriminant function analyses with and without shrinkage were similar (70.9% compared to 78.7%).

Training dataset	F	df	P	Self-test % correct	Cross-validation	Fossil mean posterior probability of group (colour) membership
Dataset 1 (ds1): black, brown, rod iridescence, grey, platelet iridescence	16.4	4,199	<0.001	77.0%	81.8%	77.6%
Dataset 2 (ds2): black, brown, rod iridescence, grey, hummingbird platelet iridescence, other platelet iridescence	15.0	5,198	<0.001	75.0%	82.5%	78.1%
Dataset 3 (ds3): black, brown, grey, hummingbird platelet iridescence, other iridescence (rod or narrow platelets in swifts, trumpeter)	24.0	4,199	<0.001	80.0%	83.0%	78.7%
Dataset 4 (ds4): black, brown, grey, rod iridescence, platelet iridescence	16.4	4,199	<0.001	77.0%	81.8%	78.1%

(fossil samples with SEM evidence for platelet morphology excluded)						
Dataset 5 (ds5): black, brown, grey, rod iridescence, platelet iridescence, penguin black colours [31] (fossil samples with SEM evidence for platelet morphology excluded)	13.5	5,198	<0.001	73.5%	82.8%	69.7%
Dataset 6 (ds6): black, brown, rod iridescence, grey, platelet iridescence (accounting for 20% shrinkage)	24.0	4,199	<0.001	77.0%	81.8%	70.9%

Supplementary Table 4: Colour reconstructions for microbodies sampled at 66 feather locations in *Caihong*. Colours predicted using canonical discriminant function analyses with six different training datasets (see Supplementary Table 3 for dataset descriptions: ds1-ds6) ID: fossil sample location (see Supplementary Fig. 6), length: microbody length (nm), diam: microbody diameter (nm). Cells show colour classification (blc: black, brw: brown, gry: grey, png: penguin brown-black colour, plt: platelet iridescence, hmm: hummingbird-like platelet iridescence) and posterior probabilities, or whether classification is based on assessment of SEM images ("SEM"), in parentheses. In some cases, both platelet and non-platelet morphologies were observed in the same sample (indicated by an *). Assuming 20% taphonomic shrinkage resulted in fewer samples predicted as black (19 versus 40) or brown (0 versus 5), and more samples predicted as showing "platelet iridescence" (24 versus 8).

ID	Length	Diam	ds1	ds2	ds3	ds4	ds5	ds6
1	1112	525	blc (0.85)	blc (0.71)	blc (0.71)	plt (SEM)	plt (SEM)	plt (0.90)
2	889	384	blc (0.77)	blc (0.76)	blc (0.76)	blc (0.80)	blc (0.62)	blc (0.59)
4	1084	502	blc (0.92)	blc (0.83)	blc (0.83)	plt (SEM)	plt (SEM)	plt (0.68)
5	778	347	brw (0.91)	brw (0.91)	brw (0.91)	brw (0.74)	brw (0.86)	blc (0.96)
6	1061	534	blc (0.90)	blc (0.88)	blc (0.88)	plt (SEM)	plt (SEM)	plt (0.78)
8	1204	501	blc (0.52)	hmm (0.71)	plt (0.71)	plt (SEM)	plt (SEM)	plt (0.77)
9	934	417	blc (0.75)	blc (0.74)	blc (0.74)	blc(0.89)*	gry (0.47)*	plt (0.45)
1	1019	476	blc	blc (0.94)	blc	plt (SEM)	plt (SEM)	plt

0			(0.97)		(0.94)			(0.68)
1			blc		blc	blc(0.42)	gry	plt
1	1216	461	(0.93)	blc (0.94)	(0.94)	*	(0.56)*	(1.00)
1			blc		blc		png	gry
2	1027	329	(0.91)	blc (0.95)	(0.93)	blc (0.93)	(0.73)	(0.52)
1			blc		blc			blc
3	895	281	(0.51)	blc (0.53)	(0.48)	blc (0.48)	blc (0.52)	(0.53)
1			plt	hmm	plt			plt
4	1132	604	(0.36)	(0.82)	(0.82)	plt (SEM)	plt (SEM)	(0.94)
1			blc		blc			plt
5	1048	544	(0.85)	blc (0.80)	(0.80)	plt (SEM)	plt (SEM)	(0.81)
1			blc		blc			plt
6	1083	552	(0.83)	blc (0.75)	(0.75)	plt (SEM)	plt (SEM)	(0.88)
1			plt	hmm	plt			plt
7	1176	625	(0.74)	(0.98)	(0.98)	plt (SEM)	plt (SEM)	(0.96)
1			plt	hmm	plt			plt
8	1192	569	(0.53)	(0.88)	(0.88)	plt (SEM)	plt (SEM)	(0.89)
1			blc		blc	brw		gry
9	889	294	(0.59)	blc (0.61)	(0.58)	(0.42)	blc (0.48)	(0.51)
2			blc	hmm	plt			plt
0	1085	596	(0.53)	(0.62)	(0.62)	plt (SEM)	plt (SEM)	(0.92)
2			blc		blc			plt
1	1135	507	(0.81)	blc (0.49)	(0.49)	plt (SEM)	plt (SEM)	(0.74)
2			blc	hmm	plt			plt
2	1085	589	(0.59)	(0.76)	(0.76)	plt (SEM)	plt (SEM)	(0.92)
2			blc		blc			plt
3	1186	461	(0.71)	blc (0.49)	(0.49)	plt (SEM)	plt (SEM)	(0.67)
2			blc		blc	blc(0.95)	gry	gry
4	1037	443	(0.96)	blc (0.90)	(0.90)	*	(0.44)*	(0.50)

2			blc		blc			plt
5	1018	345	(0.92)	blc (0.77)	(0.93)	blc (0.69)	blc (0.76)	(0.87)
2			blc		blc			blc
6	1019	311	(0.88)	blc (0.50)	(0.88)	blc (0.91)	blc (0.93)	(0.46)
2			blc		blc			gry
7	923	286	(0.69)	blc (0.72)	(0.67)	blc (0.36)	blc (0.45)	(0.58)
2			blc		blc		png	blc
8	898	323	(0.57)	blc (0.57)	(0.58)	blc (0.96)	(0.89)	(0.82)
3			blc		blc			plt
1	1104	437	(0.96)	blc (0.93)	(0.93)	blc (0.65)	blc (0.66)	(0.85)
3			blc	hmm	plt			plt
2	1168	558	(0.46)	(0.77)	(0.77)	plt (SEM)	plt (SEM)	(0.88)
3			blc		blc		png	plt
3	1087	425	(0.94)	blc (0.92)	(0.92)	blc (0.88)	(0.43)	(0.58)
3			blc		blc			gry
4	1008	316	(0.87)	blc (0.90)	(0.88)	blc (0.90)	blc (0.71)	(0.44)
3			blc		blc	blc(0.75)	gry	plt
8	1105	454	(0.92)	blc (0.77)	(0.77)	*	(0.75)*	(0.55)
4			brw	brw	brw	brw	brw	blc
0	883	331	(0.52)	(0.52)	(0.52)	(0.53)	(0.50)	(0.74)
4			blc	hmm	plt			plt
2	1112	589	(0.52)	(0.81)	(0.81)	plt (SEM)	plt (SEM)	(0.92)
4			blc		blc			plt
3	1137	448	(0.96)	blc (0.95)	(0.95)	plt (SEM)	plt (SEM)	(0.99)
4			blc		blc		gry	plt
6	1068	476	(0.96)	blc (0.89)	(0.89)	blc (0.93)	(0.52)	(0.64)
4			blc		blc			blc
7	929	352	(0.93)	blc (0.93)	(0.93)	blc (0.97)	blc (0.77)	(0.86)
4	864	362	blc	blc (0.59)	blc	blc (0.78)	blc (0.68)	blc

8			(0.60)		(0.59)			(0.50)
4			blc		blc			blc
9	952	323	(0.75)	blc (0.52)	(0.76)	blc (0.83)	blc (0.84)	(0.76)
5			blc		blc			blc
1	873	369	(0.74)	blc (0.73)	(0.73)	blc (0.84)	blc (0.73)	(0.57)
5			blc		blc		png	blc
2	973	354	(0.87)	blc (0.87)	(0.88)	blc (0.91)	(0.90)	(0.43)
5			blc		blc		brw	blc
3	847	367	(0.60)	blc (0.59)	(0.59)	blc (0.79)	(0.50)	(0.94)
5			blc		blc		png	blc
4	923	341	(0.69)	blc (0.68)	(0.69)	blc (0.79)	(0.77)	(0.62)
5			brw	brw	brw	brw	brw	blc
5	685	351	(0.94)	(0.94)	(0.94)	(0.85)	(0.95)	(0.93)
5			blc		blc		png	blc
6	917	373	(0.74)	blc (0.73)	(0.73)	blc (0.89)	(0.91)	(0.59)
5			blc		blc			gry
7	972	338	(0.87)	blc (0.83)	(0.89)	blc (0.90)	blc (0.72)	(0.59)
5			brw	brw	brw	brw	brw	blc
8	802	332	(0.89)	(0.89)	(0.89)	(0.76)	(0.79)	(0.98)
5			brw	brw	brw	brw	brw	blc
9	764	361	(0.84)	(0.84)	(0.84)	(0.52)	(0.72)	(0.88)
6			blc		blc			blc
0	928	300	(0.72)	blc (0.76)	(0.68)	blc (0.83)	blc (0.86)	(0.74)
6			blc		blc			gry
1	973	277	(0.79)	blc (0.84)	(0.74)	blc (0.70)	blc (0.67)	(0.54)
6			blc		blc			plt
3	1032	356	(0.94)	blc (0.93)	(0.95)	blc (0.96)	blc (0.62)	(0.48)
6			blc		blc			blc
4	1050	277	(0.87)	blc (0.90)	(0.82)	blc (0.87)	blc (0.89)	(0.42)

6			blc		blc			gry
5	1026	301	(0.87)	blc (0.90)	(0.85)	blc (0.87)	blc (0.59)	(0.44)
6			blc		blc			gry
6	1045	301	(0.87)	blc (0.91)	(0.84)	blc (0.89)	blc (0.92)	(0.43)

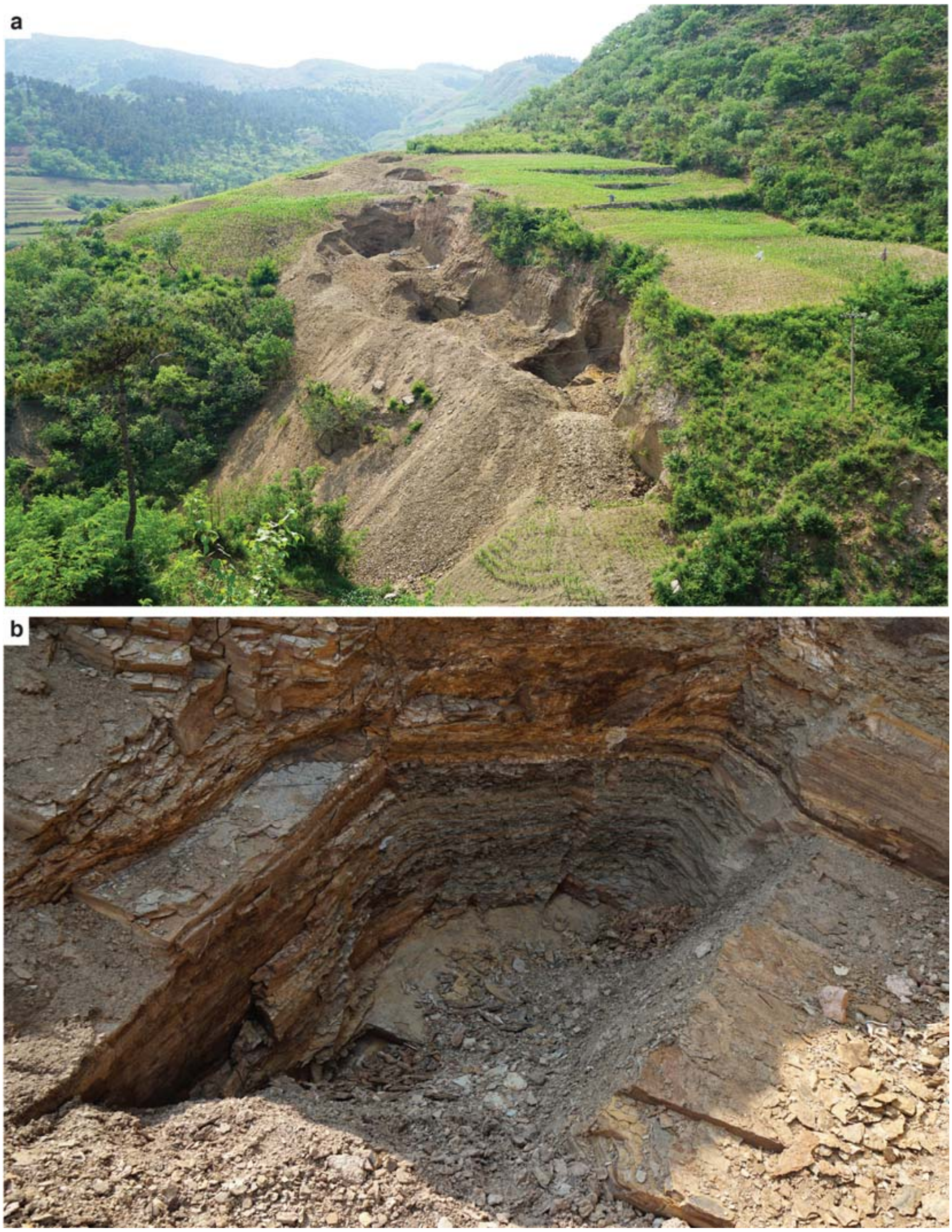

Supplementary Figure 1. Nanshimen Locality in Gangou Township, Qinglong County, Hebei. The quarries near the Nanshimenzi Village and Nanshimencun Village are generally called Nanshimen Locality (also called Zhuanshanzi Locality or Gangou Locality in some literature) and they produced numerous vertebrate fossils including the theropod *Yi qi*. The lower image shows an excavation we organized at one of these quarries near the Nanshimenzi Village in the summer of 2016 in order to confirm the provenance of PMoL-B00175.

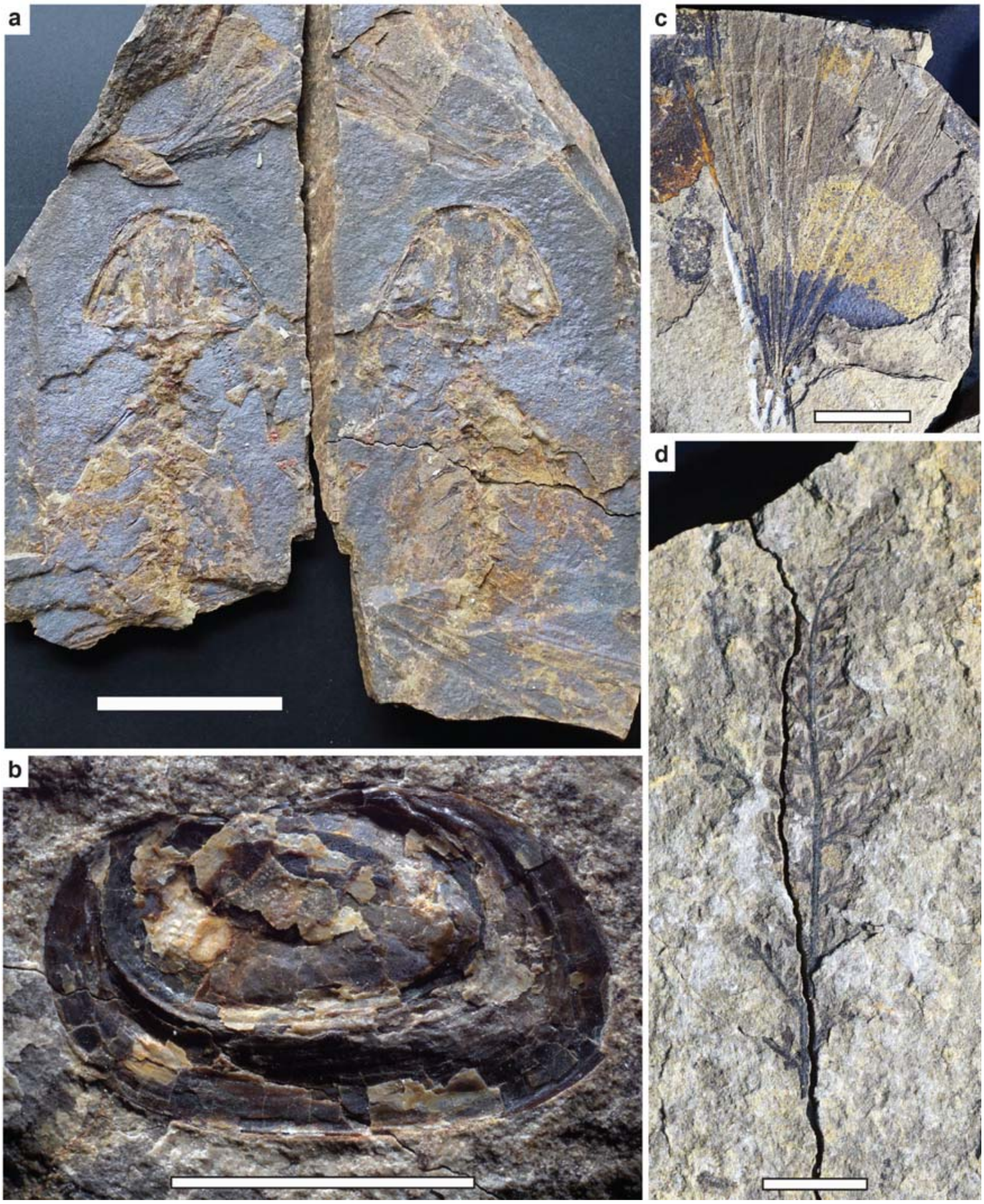

Supplemenary Figure 2. Photographs of newly collected fossils from the Nanshimen Locality. (a) The salamander *Qinglongtriton gangouensis*; (b) the bivalve *Arguniella* sp; (c) the plant *Czekanowskia* sp; (d) the plant *Coniopteris hymenophylloides*. Scale bars: 5 cm in (a), 2 cm in (b), 0.5 cm in (c, d).

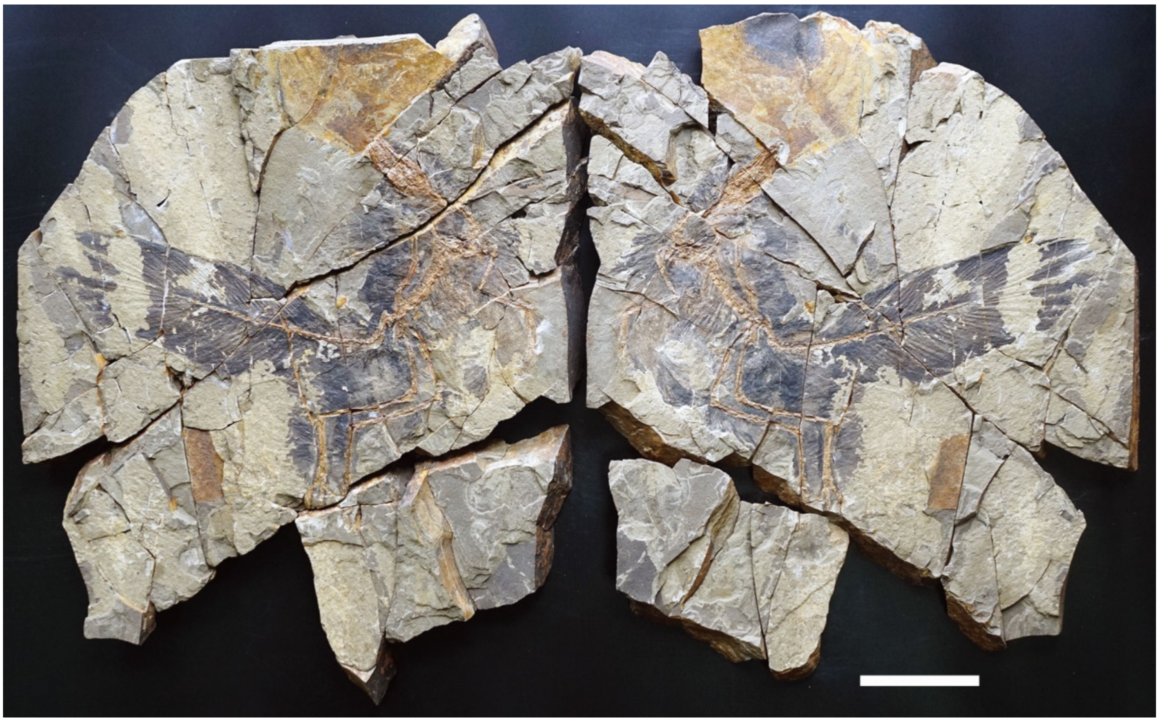

Supplementary Figure 3. Photograph of the PMoL-B00175. The slab (left) and counter slab (right) mirror to each other. Scale bar: 10 cm

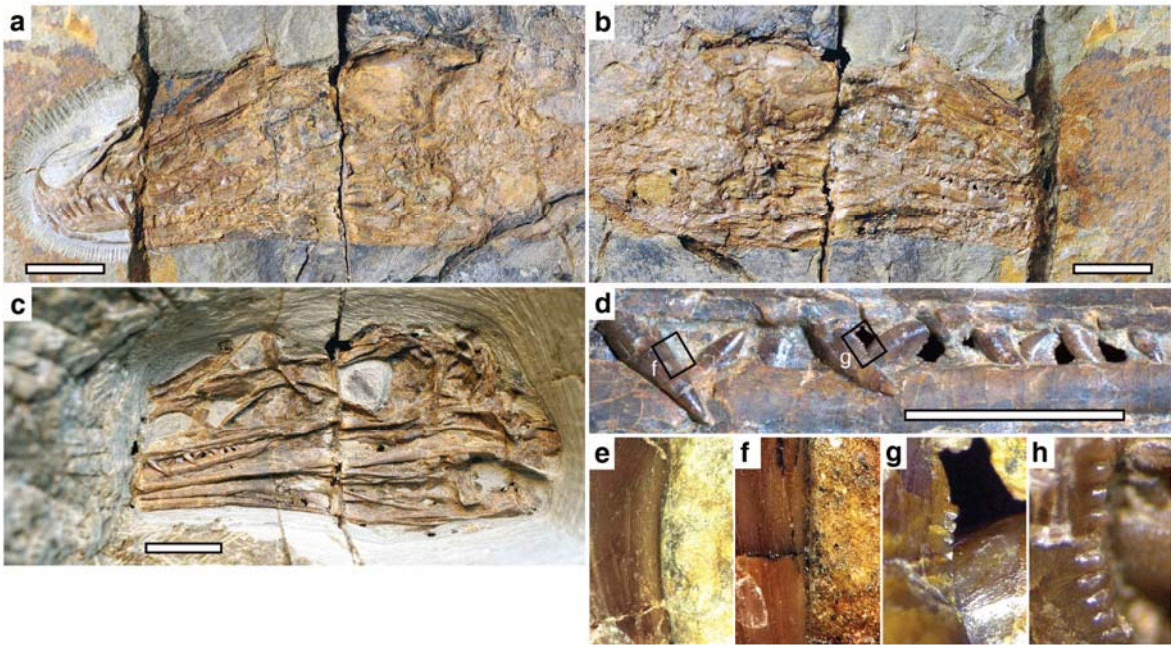

Supplementary Figure 4. Photographs of the skull and mandible of the PMoL-B00175. (a) The skull and mandible exposed on the counter slab; (b) the skull and mandible exposed on the slab; (c) the skull and mandible exposed from the bottom side of the slab; (d) mid-posterior teeth in lateral view; close-up of posterior margins of the 3rd (e), 4th (f), 5th (g) maxillary tooth and a dentary tooth (f). Scale bars: 1 cm in (a, b, c), 0.5 cm in (d); photos e, f, g and h with a height of 1 mm; photos f and g are close-up of the area in the rectangles in (d).

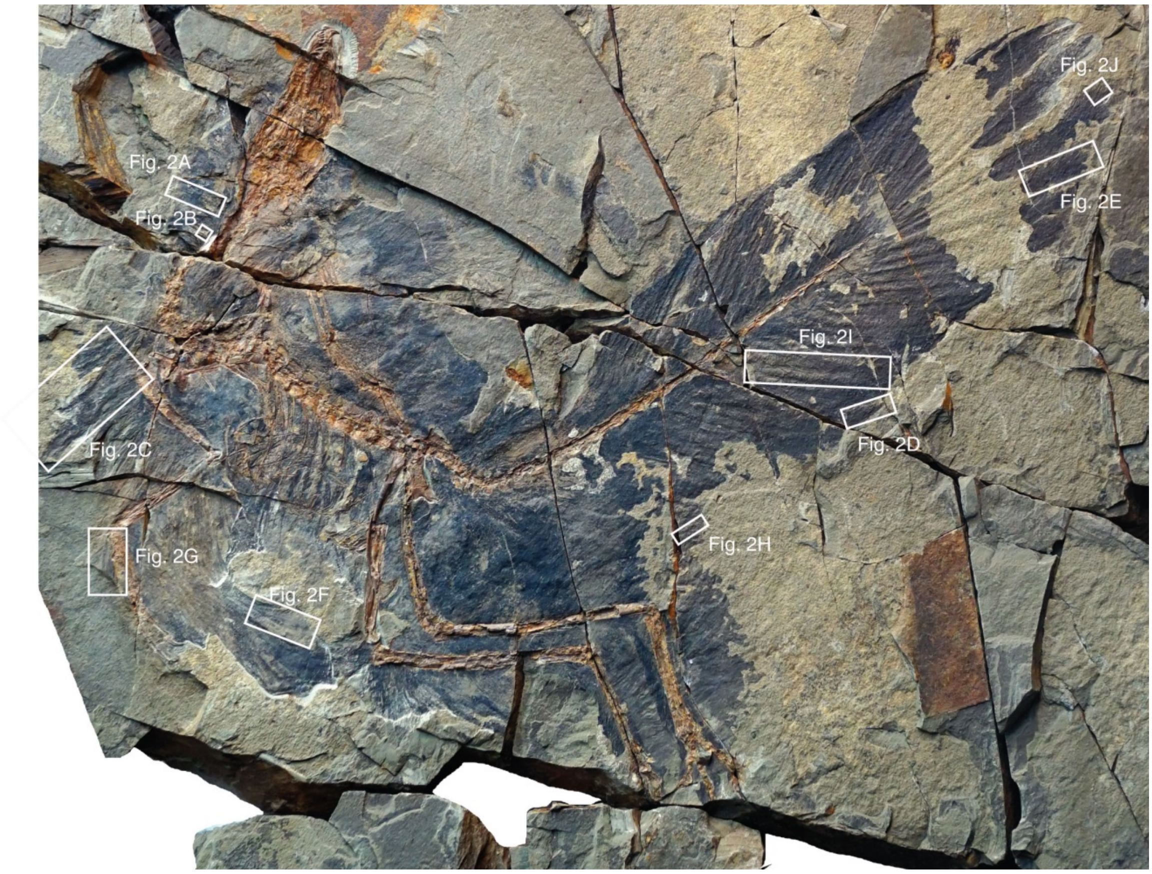

Supplementary Figure 5. Locations of feathers illustrated in Fig. 4.

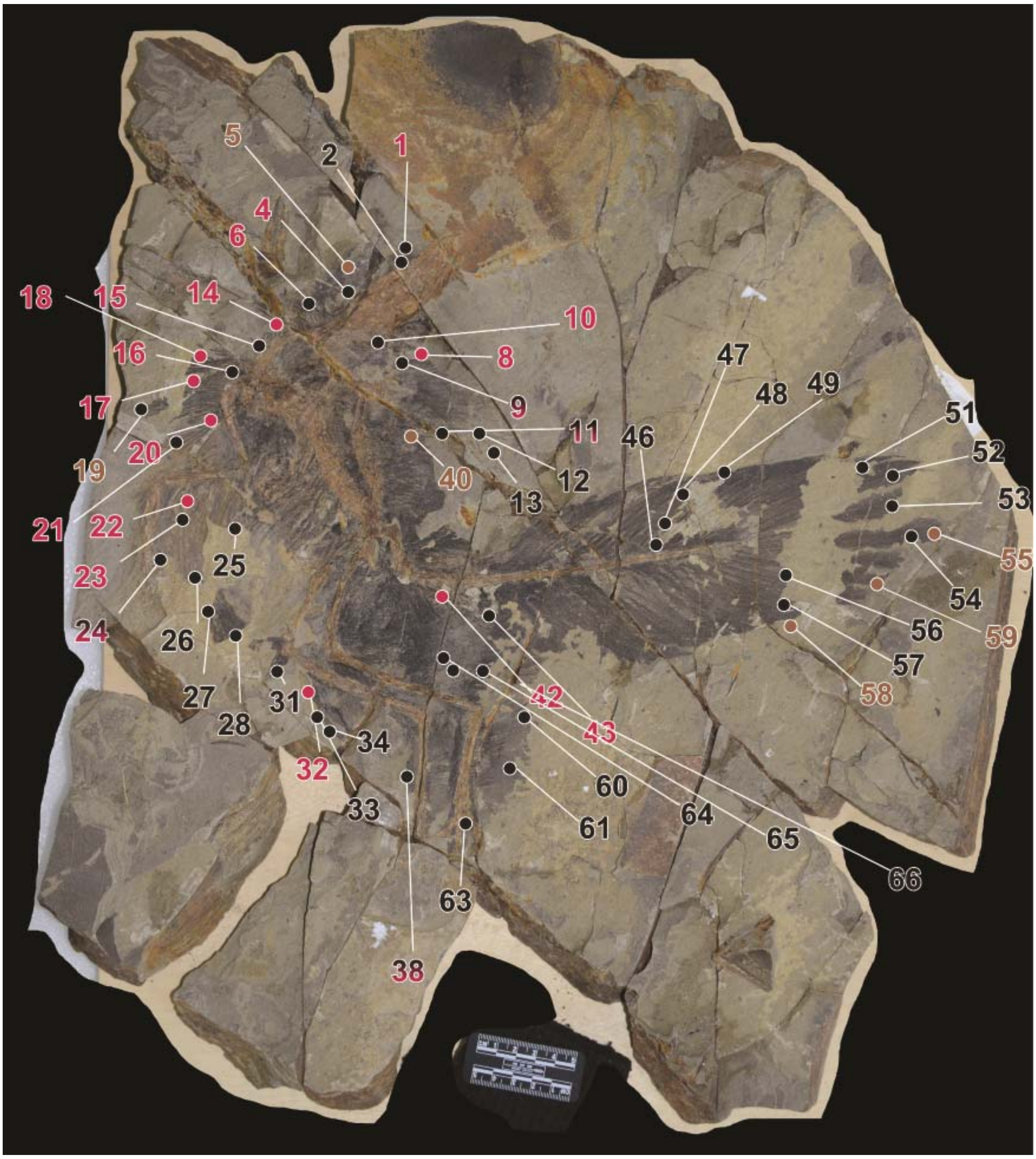

Supplementary Figure 6. Microbody sampling locations and colour reconstruction in *Caihong juji*. Text colours correspond to colour reconstructed based on combined assessment of SEM images (Supplementary Fig. 7) and quadratic discriminant analysis (black: black, grey: grey, brown: brown, red: platelet iridescence). Point colours show predictions from quadratic discriminant analysis using only microbody measurements. Samples with both platelet-shaped and elliptical microbodies are represented by a vertical colour gradient. Measurements taken near internal organs/body cavity (e.g., samples 35, 36, 39, 41) were excluded from the dataset prior to analyses, resulting in a final dataset of 53 locations in the fossil slab.

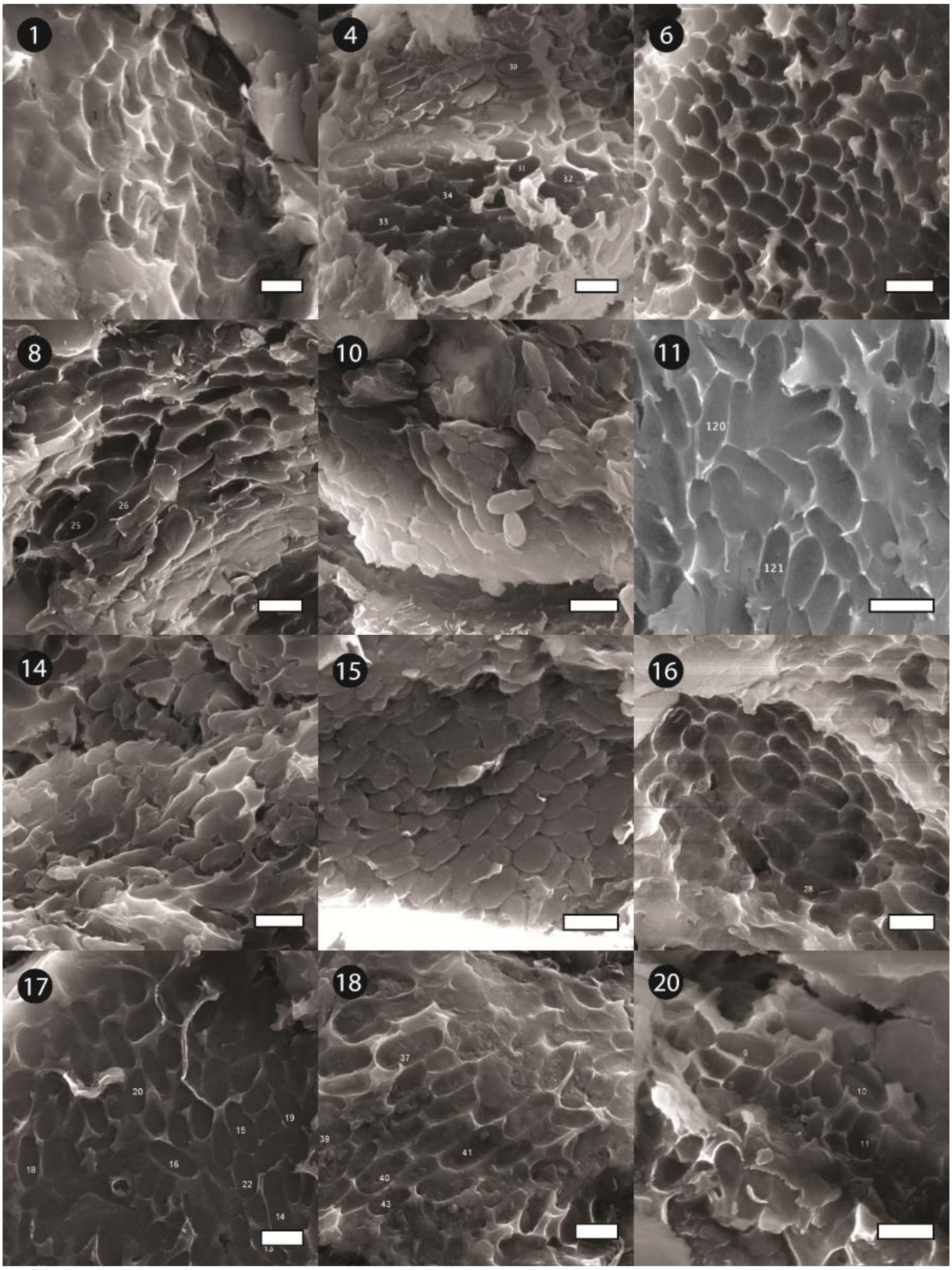

1
4
30
31
32
34
33
6
8
26
25
10
11
120
121
14
15
16
28
17
20
19
15
18
16
22
14
18
37
39
41
40
43
20
9
10
11

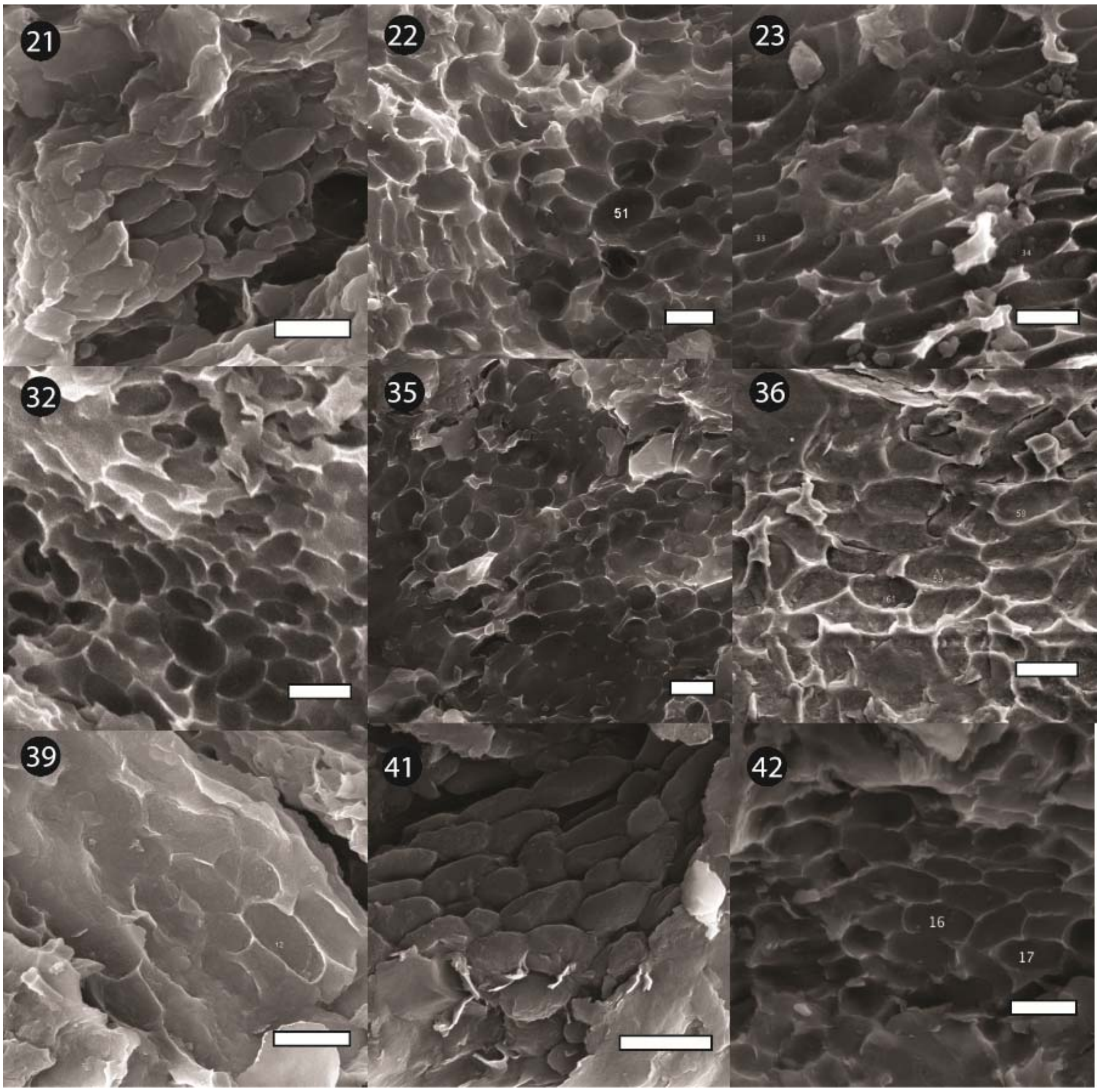

Supplementary Figure 7. Representative SEM images showing evidence for platelet-like microbodies in *Caihong juji*. Evidence for flat, platelet-like microbodies included (i) flat, platelet-shaped 3D shapes (e.g., 4, 41), (ii) overlapping microbody molds (e.g., 6, 16, 22, 42), and iii) dense packing, vertical stacking (e.g., 41) and sheet-like arrangement of microbodies (e.g., 6, 15). Panel numbers correspond to sample locations in Supplementary Fig. 6. All scale bars are 1000 nm.

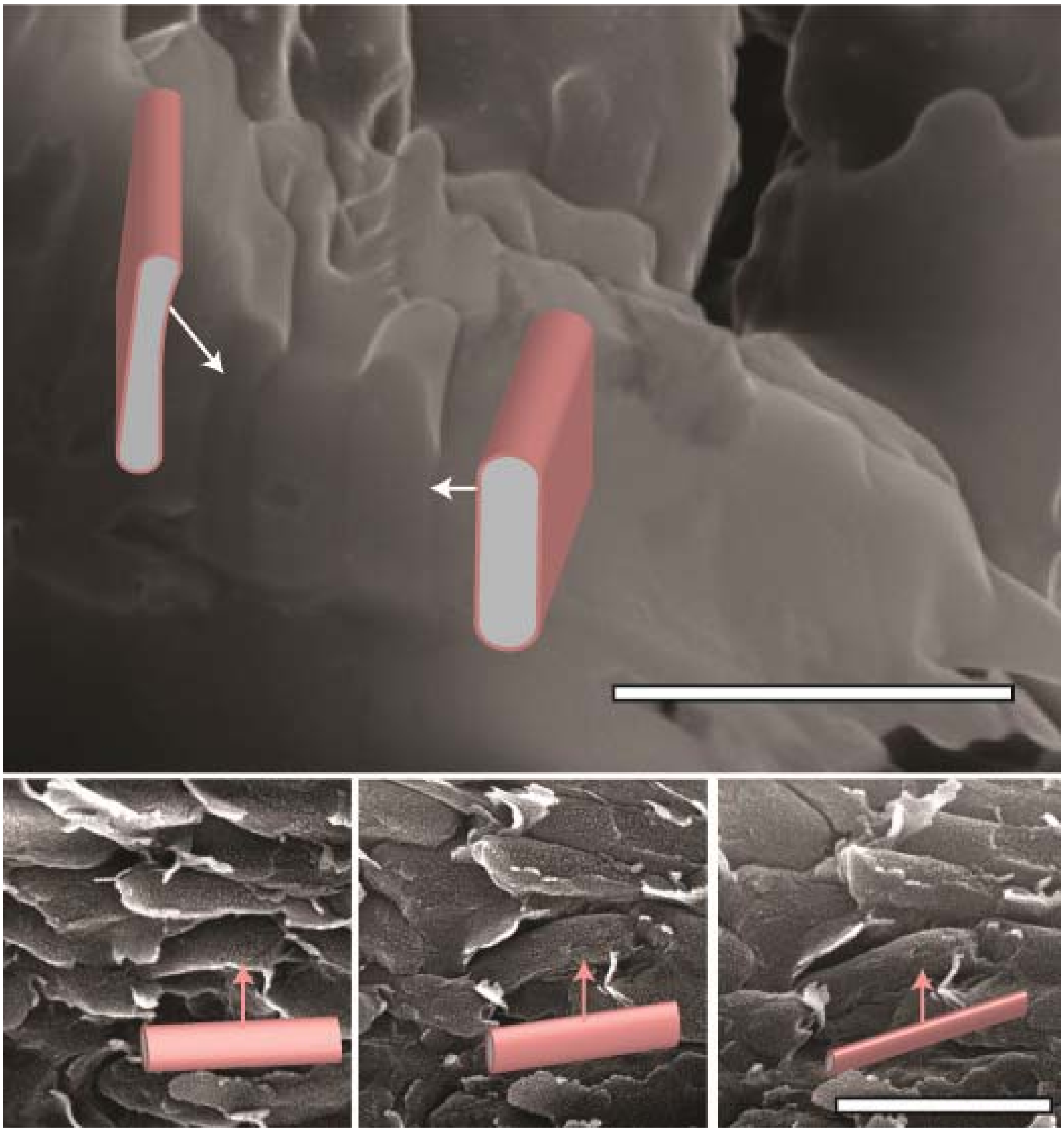

Supplementary Figure 8. Evidence for platelet-shaped microbodies in *Caihong juji*. Upper SEM image shows cross-section through sample 4 in Supplementary Fig. 6. Lower series of images (all at the same scale) shows same region at 0º, 45º and 55º stage rotation. Schematics depict orientation of platelets. Scale bars: 1000 nm.

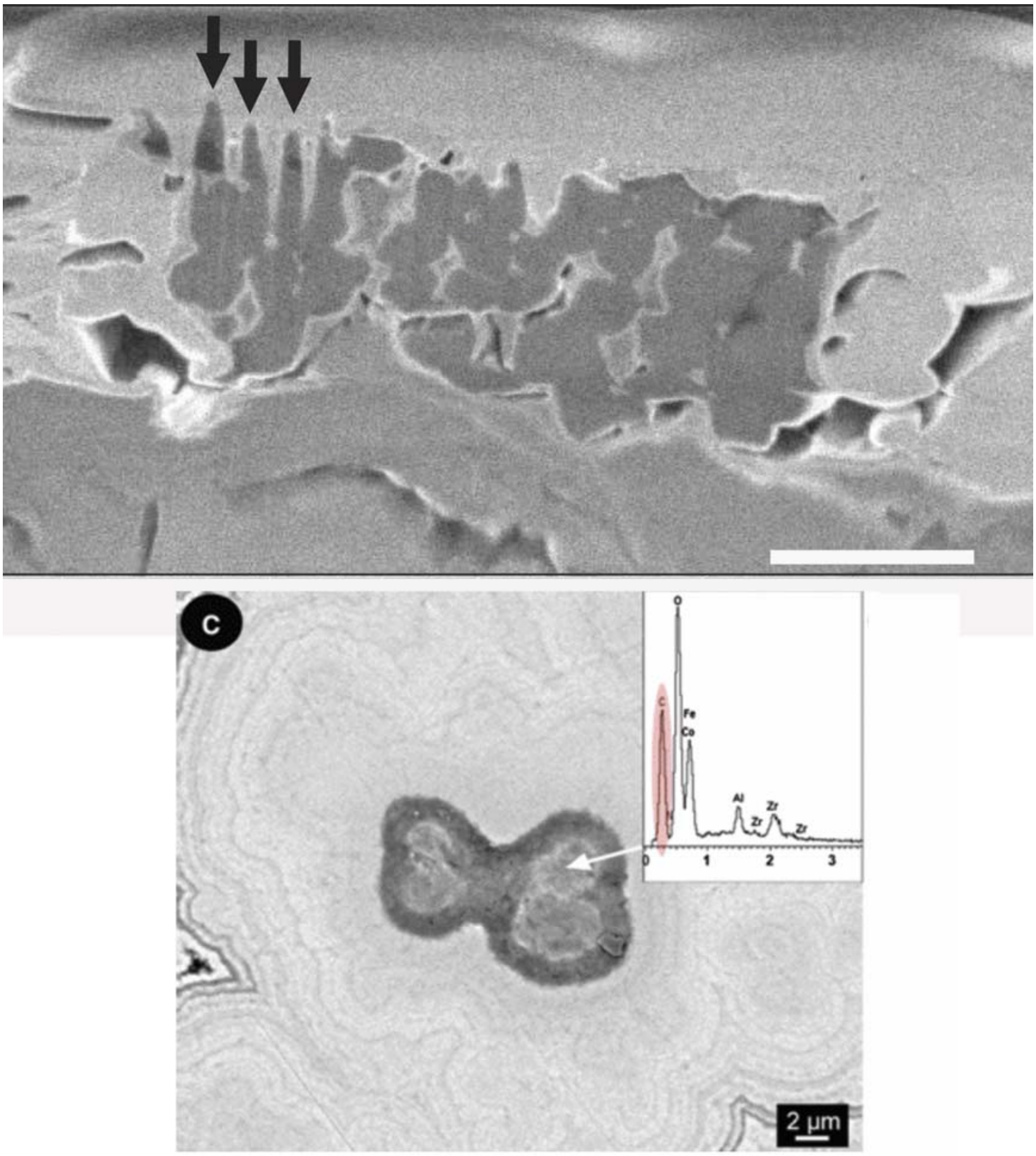

Supplementary Figure 9. Comparative FIB-SEM images of microbodies in a sample of *Caihong* and a bacterium fossil. Top: FIB-SEM cross-sectional image showing microbodies (dark, electron-dense regions) in a sample of *Caihong*. The microbodies are solid, <1um in diameter, and some (arrows) are flattened. Bottom: A fossilized microbody identified as a bacterium (Fig. 7C of Supplementary ref. [32]). Relative to the microbodies in *Caihong*, the putative bacterium is less electron-dense in the core, larger (>4 um in diameter), not flattened, and appears to be undergoing fission.

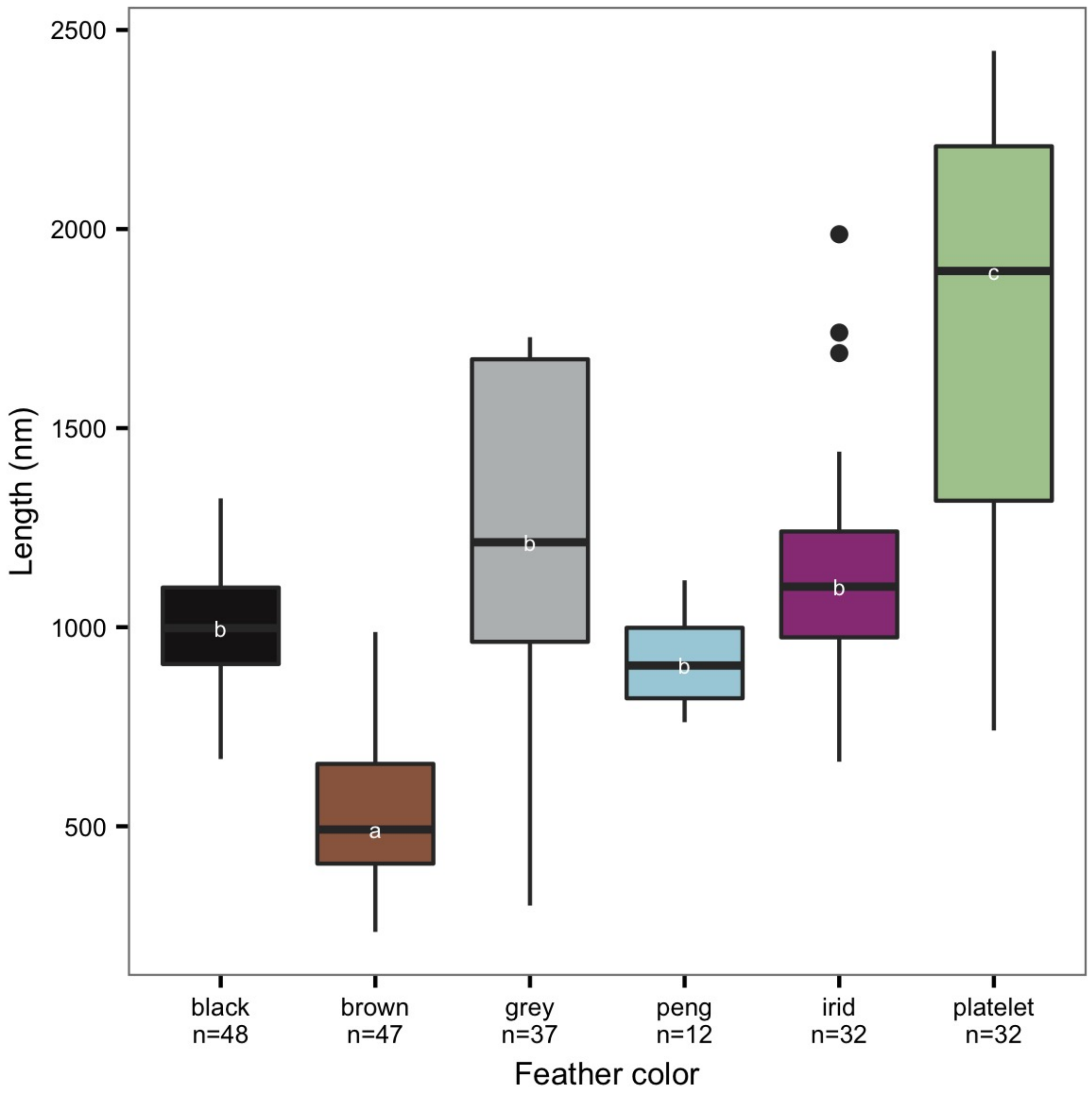

Supplementary Figure 10. Melanosome length variation in extant birds. Boxplots sharing similar letters are not significantly different (Tukey tests, $p < 0.05$). Length of melanosomes in birds with platelet-shaped melanosomes is significantly greater than all other colour classes. Melanosomes associated with brown colours are significantly shorter than all other colour classes. Overall ANOVA was highly significant ($F_{5,202} = 56.4$, $p < 0.001$).

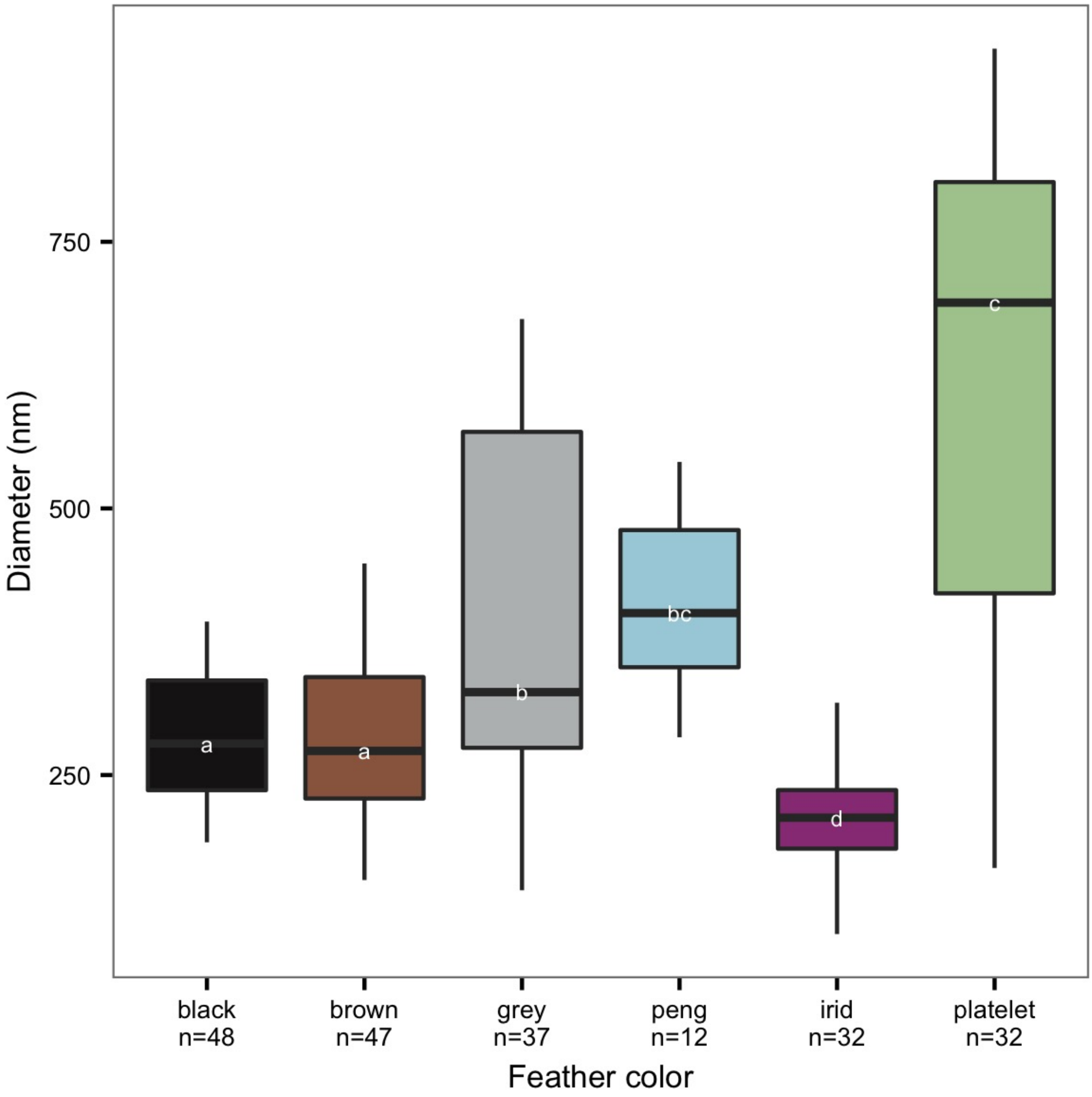

Supplementary Figure 11. Melanosome diameter variation in extant birds. Boxplots sharing similar letters are not significantly different (Tukey tests, $p < 0.05$). Width of melanosomes in birds with “platelet-type” melanosomes is significantly greater than all other colour classes except penguin melanosomes. Melanosomes associated with rod iridescence are significantly narrower than all other colour classes. Overall ANOVA was highly significant ($F_{5,202} = 33.4$, $p < 0.001$).

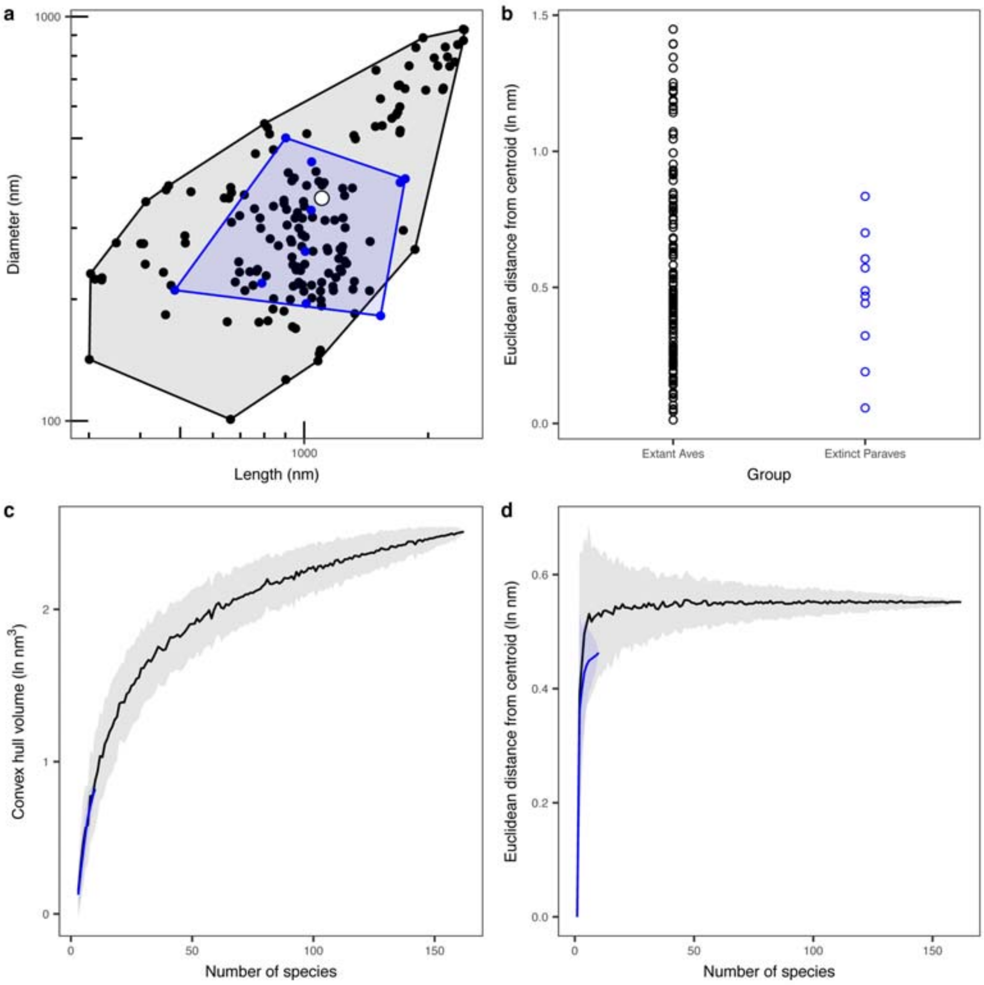

Supplementary Figure 12. Comparison of microbody diversity in feathers of extinct paravian dinosaurs and extant birds. Colours correspond to extinct feathered paravian dinosaurs (blue, n = 10) and extant birds (black, n = 162). (a) Morphospace species means for microbody diameter and length. Filled white circle is multivariate mean morphology. Shaded polygons are convex hulls. Axes log-transformed to better visualize extreme values. (b) Disparity (Euclidean distance from mean morphology) for each group. Difference was not statistically significant (two-sample *t*-test, $t = 1.06$, $p = 0.31$; Mann-Whitney test, U = 849, p = 0.80). (c, d) Rarefaction curves for convex hull volume (c) and mean Euclidean distance from centroid (d) as a function of species sample size. Disparity metrics were resampled 200 times for each number of species (shaded polygons: ± 1 SD). Convex hull volumes are still increasing at large sample sizes (n > 150 species), suggesting that further sampling will increase estimated diversity. Microbodies in extinct paravians, inferred as melanosomes, are just as diverse as in extant paravians considering the metric of Euclidean distance from the mean morphology.

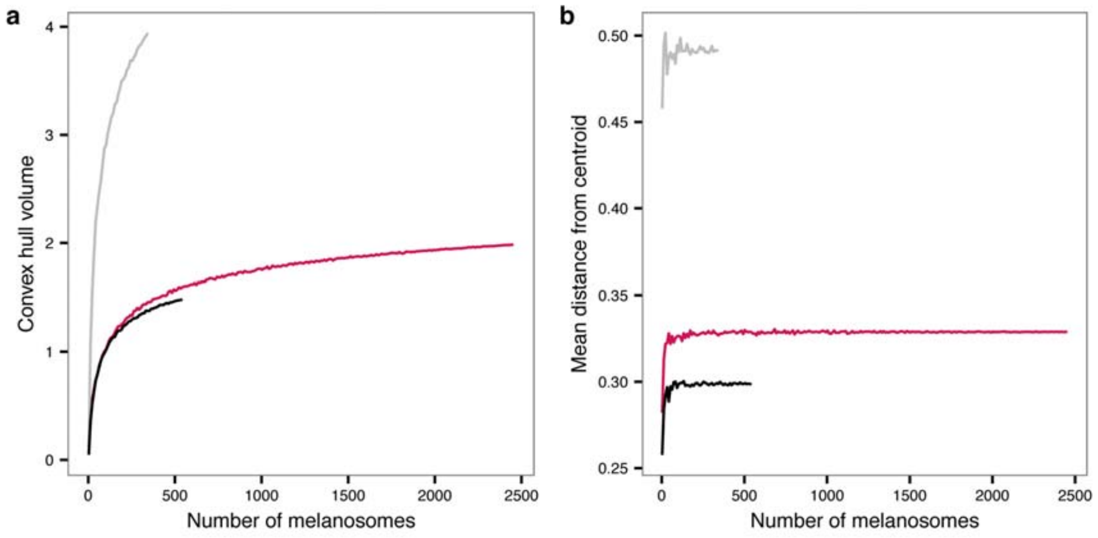

Supplementary Figure 13. Comparison of microbody diversity in sampled extinct paravians. Rarefaction curves showing convex hull volume (a) and mean Euclidean distance from centroid (b) as a function of species sample size. Mean values for each disparity metric (solid lines) were computed 200 times at each sample size. Colours correspond to different taxa: *Microraptor* (black), *Anchiornis huxleyi* (grey) and *Caihong juji* (dark red). The convex hull volume continually increases, suggesting that further sampling would recover even greater microbody diversity but the relative pattern (*Anchiornis* > *Caihong* and *Microraptor*) would likely stay constant. Microbody diversity as measured by the Euclidean distance from the mean group morphology (b) is adequately sampled even at relatively small (n < 300) sample sizes.

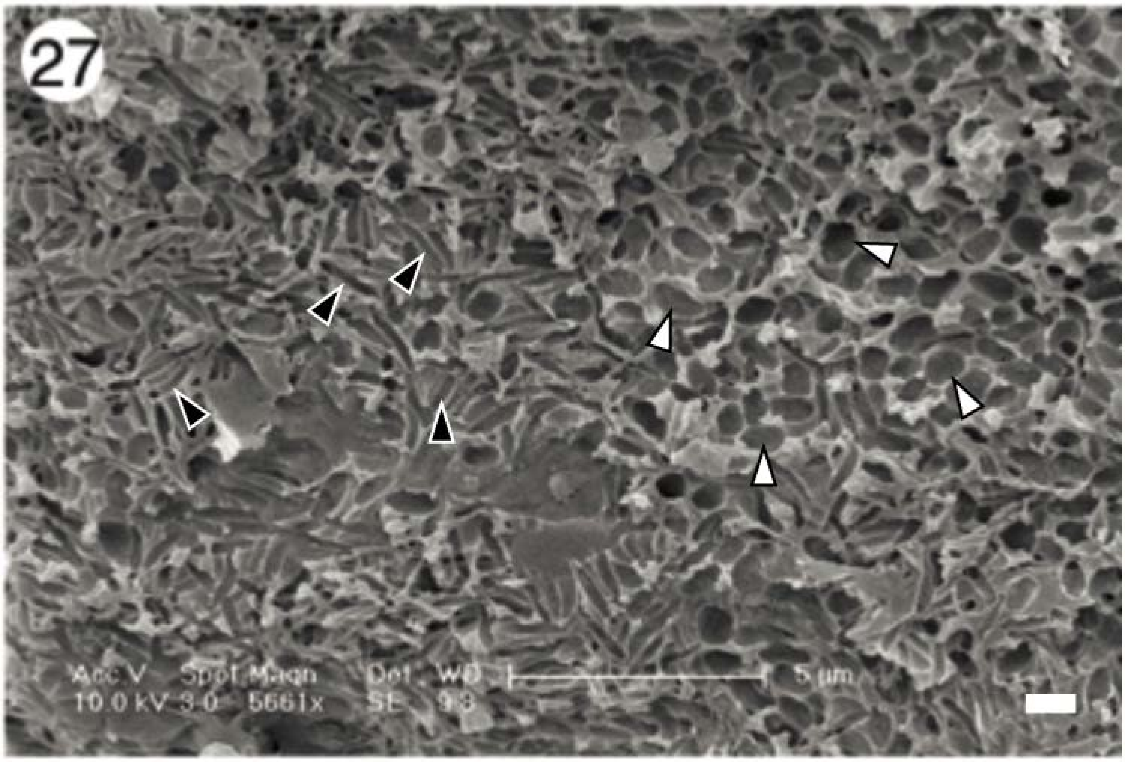

Supplementary Figure 14. Microbody impressions in the *Anchiornis huxleyi* sampled in [29] (sample 27 from the head of the animal). Annotations illustrate mouldic forms of more spherical presumed melanosomes that do not overlap (open arrowheads), unlike those observed in *Caihong juji* (Supplementary Fig. 7), along with more highly elongated microbodies (closed arrowheads). Scale bar: 1000 nm.

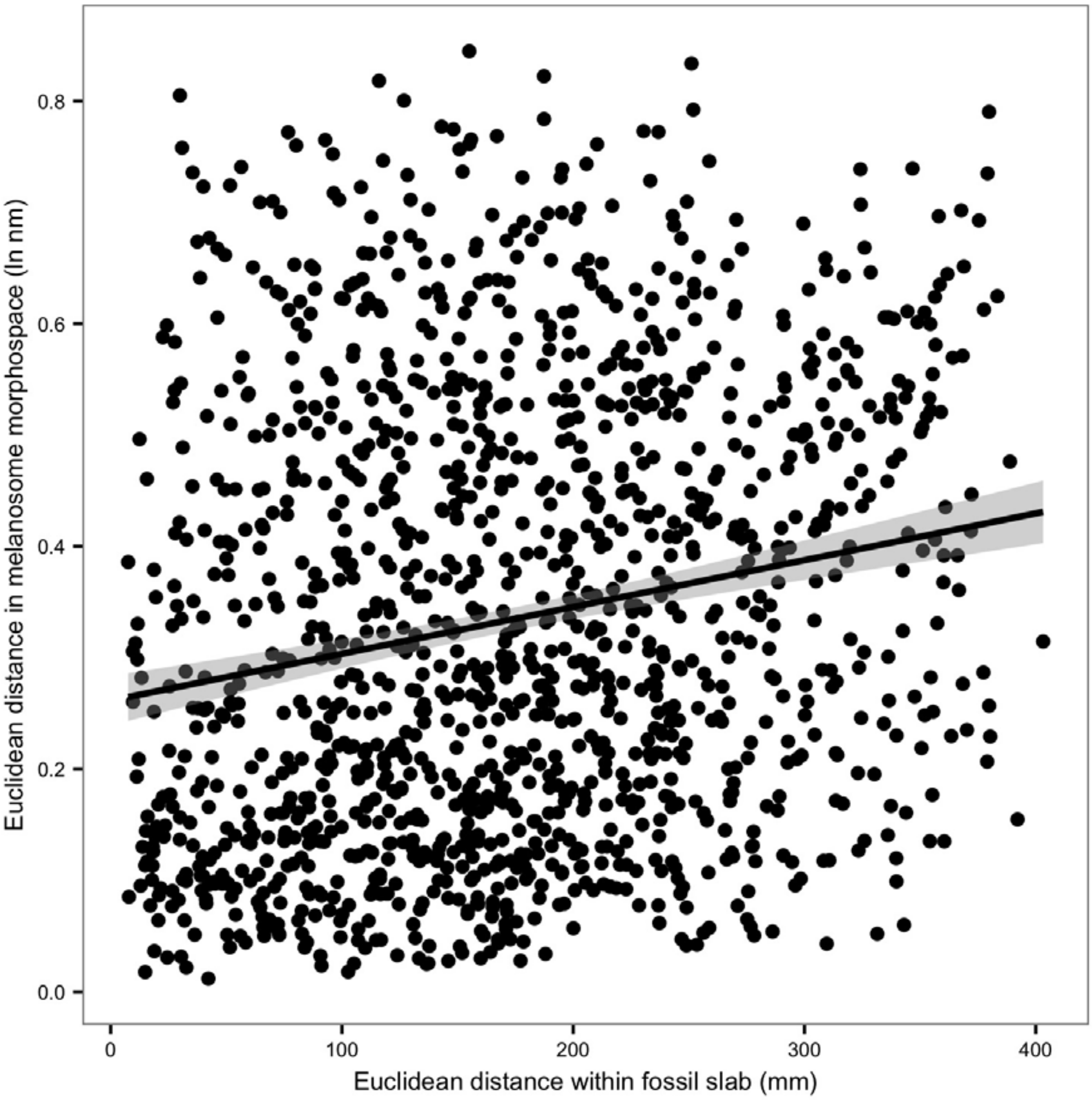

Supplementary Figure 15. *Caihong juji* microbodies sampled from nearby locations in the fossil are more morphologically similar. Mantel's test on Euclidean distance matrices showed a significant relationship between microbody shape diversity (natural log-transformed length and diameter) and spatial distance (in mm) within the fossil slab (Mantel's $r = 0.19$, $p = 0.001$). This indicates that morphology (and colour) of microbodies varies systematically across the body (i.e. variation is distributed in distinct "patches").

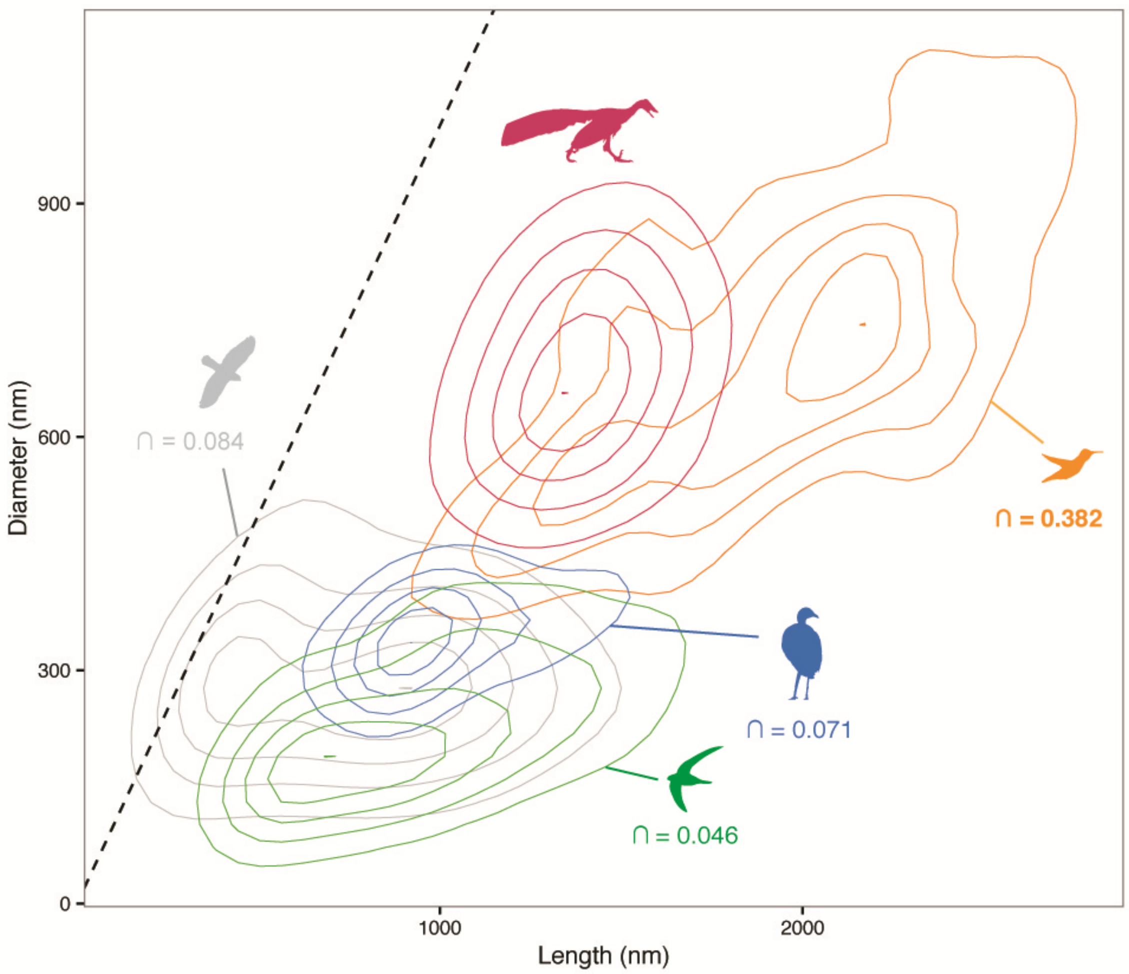

Supplementary Figure 16. Accounting for taphonomic shrinkage increases overlap between *Caihong* and extant hummingbirds. Morphospace plots of raw microbody measurements for *Caihong juji* (multiplied by 1.25 to account for 20% shrinkage in the fossil samples) relative to other extant avian groups with platelet-shaped (yellow: hummingbirds, green: swifts, blue: trumpeters and trogons) and elliptical melanosomes (grey: other extant Aves). Contour lines show 2D density of length and diameter measurements. Numbers give proportional overlap (∩) between *Caihong juji* (red) and each group. Dashed line indicates 1:1 length to diameter ratio (i.e. spherical melanosomes or microbodies). After accounting for 20% shrinkage, all extant groups except hummingbirds overlapped less in length and diameter with *Caihong*, while hummingbirds showed much stronger overlap (0.382 with compared to 0.241 without shrinkage). Photo credits (silhouettes created by C. Eliason): Alan Vernon (hawk, CC BY 2.0), T. R. Shankar Raman (swiftlet, CC BY-SA 4.0), Dick Daniels (trumpeter, CC BY SA 3.0), Velizar Simeonovski (*Caihong*, J. Clarke/U. Texas), Pacific Southwest Region (hummingbird, CC BY-2.0).

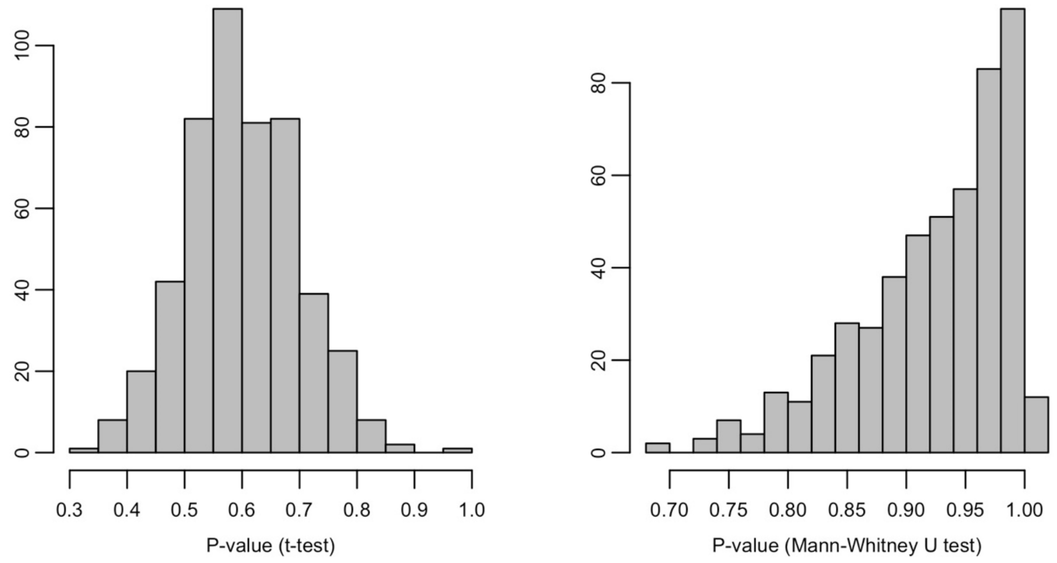

Supplementary Figure 17. Effect of among-taxon variation in sample shrinkage on the comparison of diversity in extant birds and extinct paravian dinosaur taxa. Histograms show distribution of p-values for 500 simulations of among-taxon variation in sample shrinkage, ranging from 10% [33] to 20% [34] drawn from a random uniform distribution. Results indicate that among-species variation in taphonomic shrinkage does not influence our result of similar diversity between extant birds and extinct paravian dinosaurs (Supplementary Fig. 12).

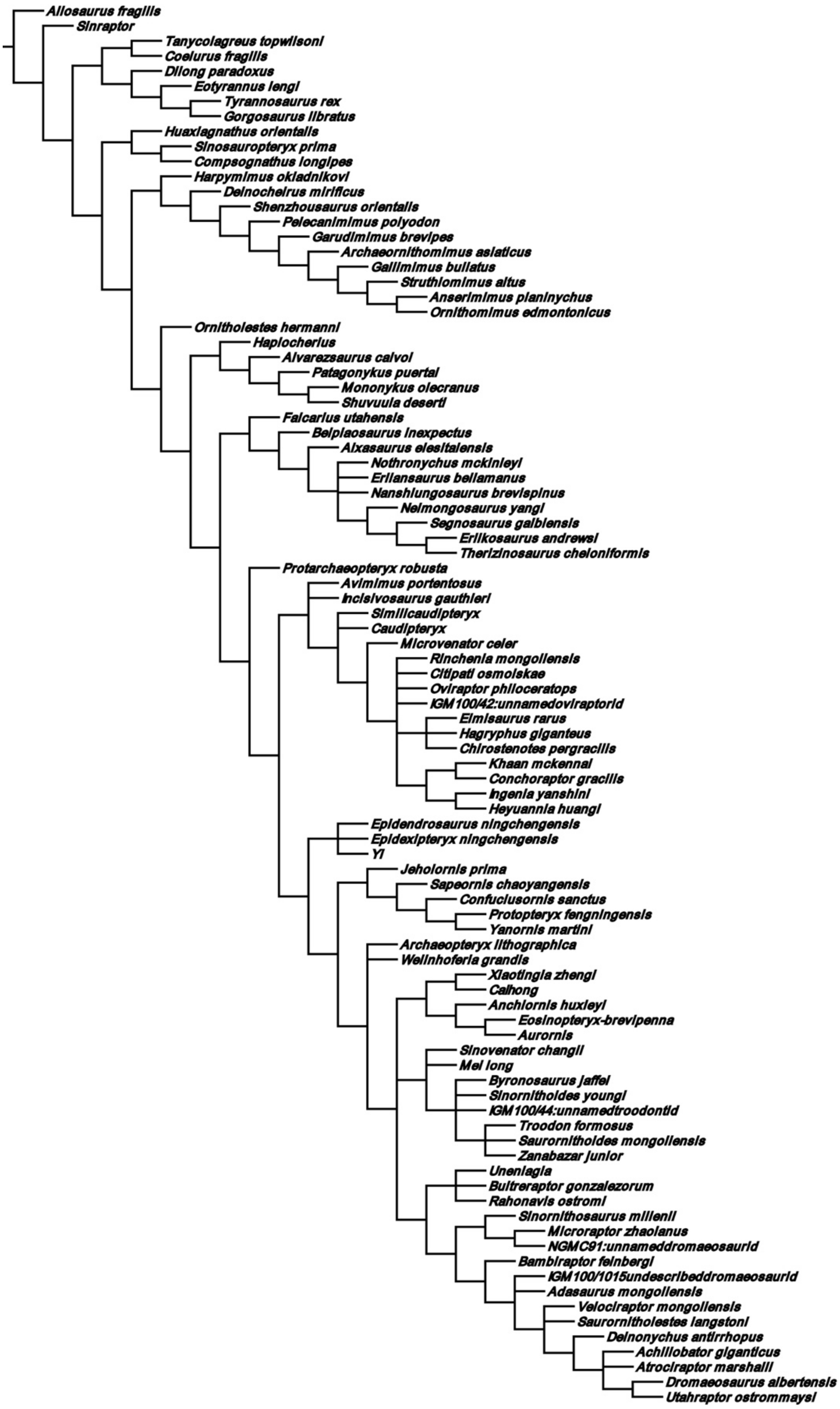

Supplementary Figure 18. Strict consensus of 192 most parsimonious trees resultant from analysis of the dataset from Supplementary ref. [8] with *Caihong* and the expanded taxonomic sample.

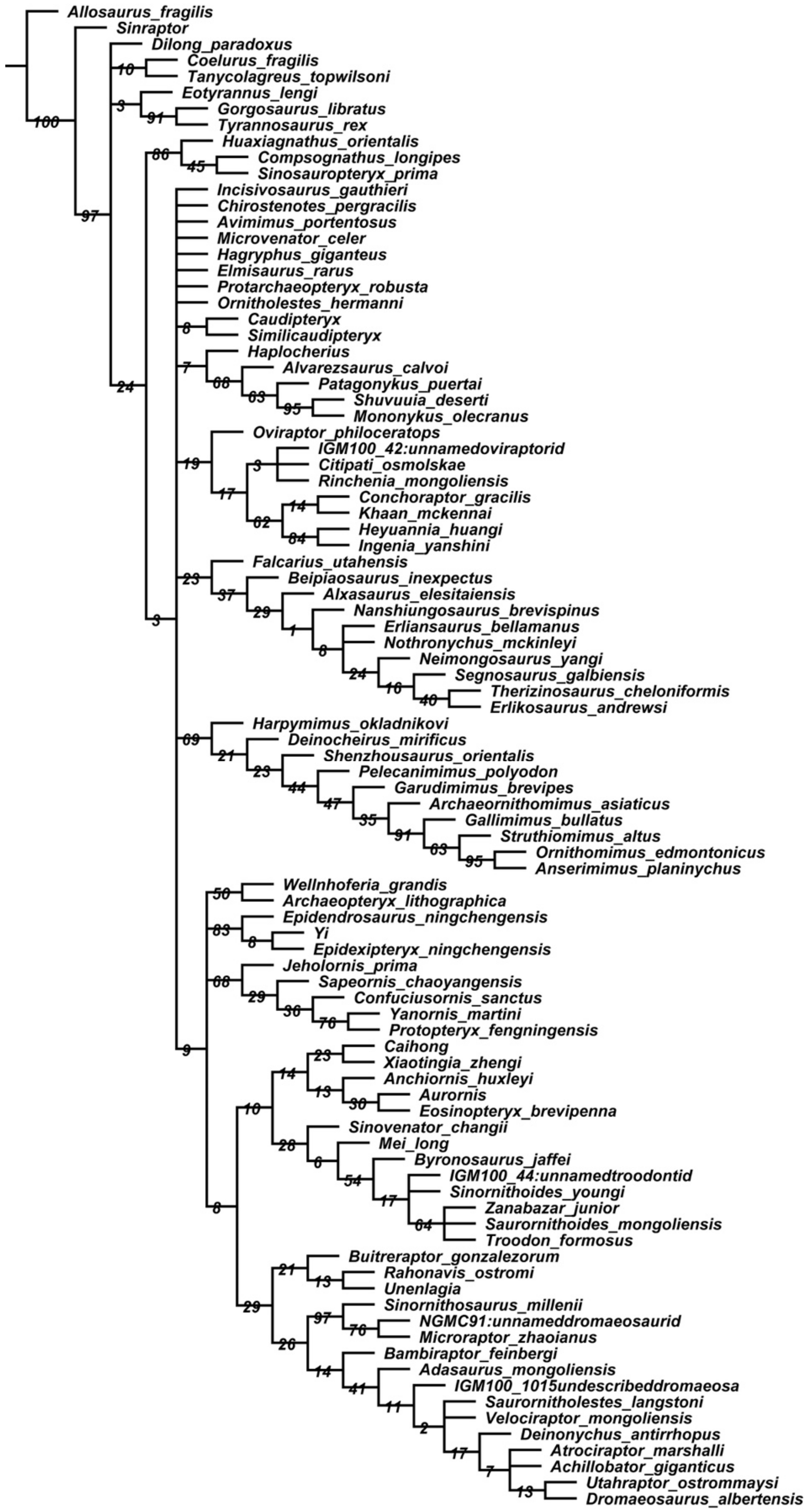

Supplementary Figure 19. Bootstrap values for the clades recovered by the analysis of the dataset from Supplementary ref. [8].

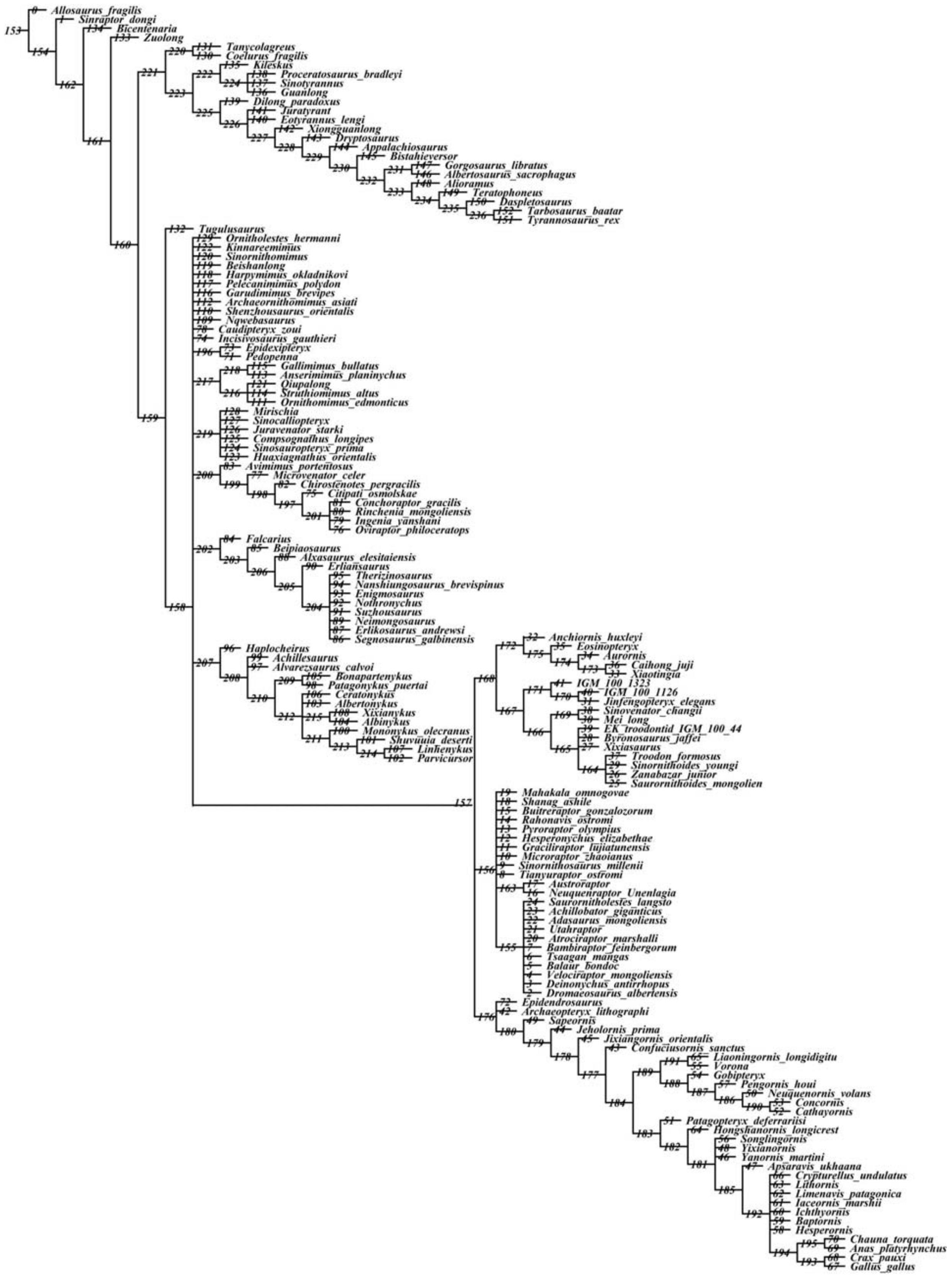

Supplementary Figure 20. The strict consensus of 100000 most parsimonious trees recovered by analysis of the dataset from Supplementary ref. [28] (all multistate characters unordered)

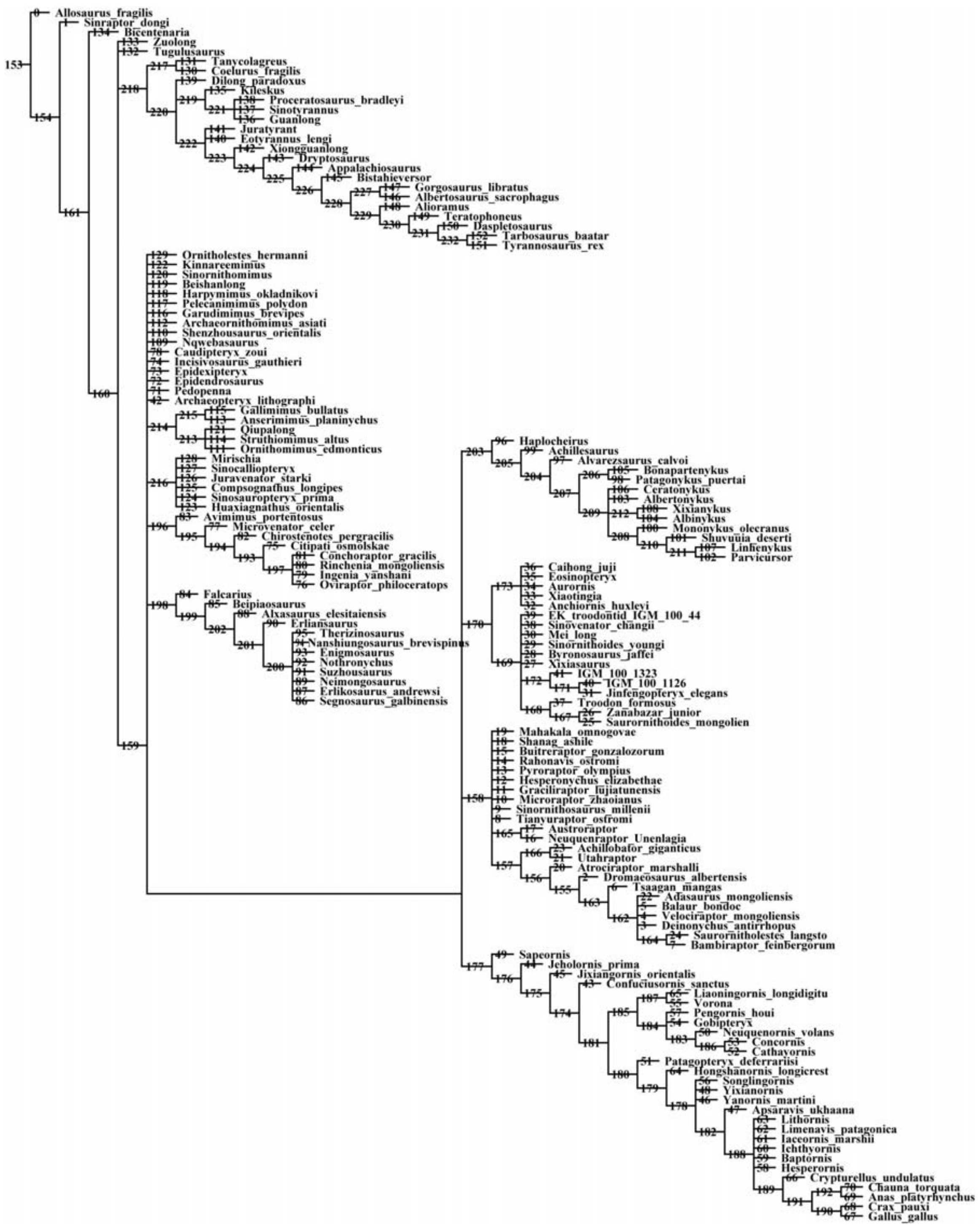

Supplementary Figure 21. The strict consensus of 100000 most parsimonious trees recovered by analysis of the dataset from Supplementary ref. 28 (some multistate characters ordered following **Supplementary ref. 28**

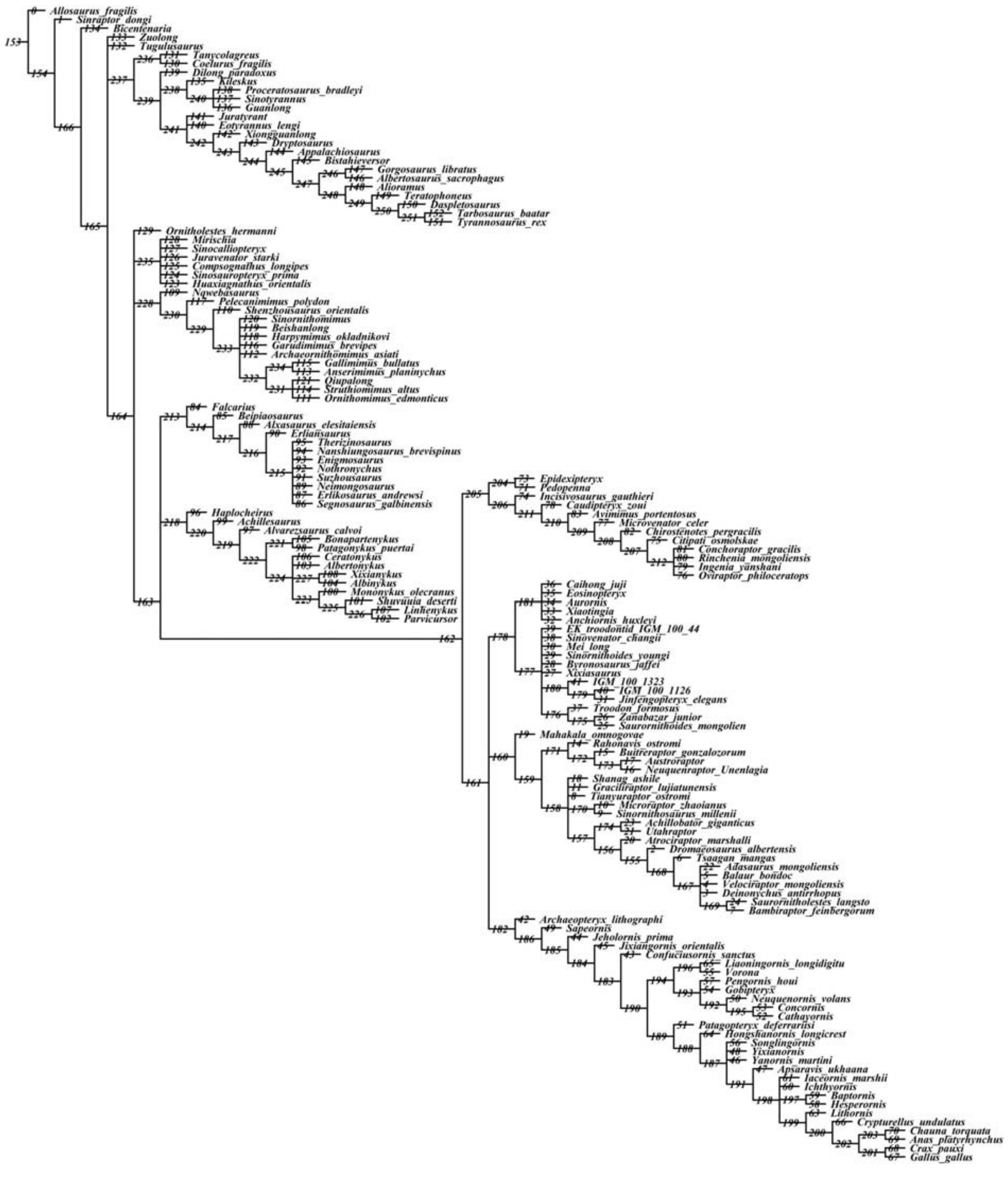

Supplementary Figure 22. Reduced strict consensus of 100000 most parsimonious trees recovered by analysis of the Brusatte matrix (some multistate characters ordered following Supplementary ref. [28] (tree length=3360 steps). The reduced strict consensus is calculated after the a posteriori removal of five taxa: *Kinnareemimus*, *Epidendrosaurus*, *Pyroraptor*, *Hesperonychus*, and *Limenavis*

Supplementary References

1. Xu K*, et al. Jurassic system in the North of China (VII): the stratigraphic region of Northeast China*. Petroleum Industry Press (2003).
2. Xu X, Zhou ZH, Sullivan C, Wang Y, Ren D. An updated review of the Middle-Late Jurassic Yanliao Biota: Chronology, Taphonomy, Paleontology, and Paleoecology. *Acta Geologica Sinica (English Edition)* **90**, 1801–1840 (2016).
3. Sullivan C, Wang Y, Hone DWE, Wang Y, Xu X, Zhang F. The vertebrates of the Jurassic Daohugou Biota of northeastern China. *Journal of Vertebrate Paleontology* **34**, 243-280 (2014).
4. Jia J, Gao K-Q. A new basal salamandroid (Amphibia,Urodela) from the Late Jurassic of Qinglong,Hebei Province, China. *PLoS ONE* **11(5)**, e0153834 (2016).
5. Lü JC. A new non-pterodactyloid pterosaur from Qinglong County, Hebei Province of China. *Acta Geologica Sinica (English edition)* **83**, 89-199 (2009).
6. Lü J, Unwin DM, Zhao B, Gao C, Shen C. A new rhamphorhynchid (Pterosauria: Rhamphorhynchidae) from the Middle/Upper Jurassic of Qinglong, Hebei Province, China. *Zootaxa* **3158**, 1–19 (2012).
7. Lü J, Hone DWE. A new Chinese anurognathid pterosaur and the evolution of pterosaurian tail lengths. *Acta Geologica Sinica (English Edition)* **86**, 1317–1325 (2012).
8. Xu X*, et al.* A bizarre Jurassic maniraptoran theropod with preserved evidence of membranous wings. *Nature* **521**, 70-73 (2015).
9. Zheng X, Bi S, Wang X, Meng J. A new arboreal haramiyid shows the diversity of crown mammals in the Jurassic period. *Nature* **500**, 199-202 (2013).
10. Huang DY. Yanliao Biota and Yanshan Movement. *Acta Palaeontologica Sinica* **54**, 501-546 (2015).
11. Zhang FC, Zhou ZH, Xu X, Wang XL, Sullivan C. A bizarre Jurassic maniraptoran from China with elongate ribbon-like feathers. *Nature* **455**, 1105-1108 (2008).
12. Hu DY, Hou L-H, Zhang LJ, Xu X. A pre-*Archaeopteryx* troodontid from China with long feathers on the metatarsus. *Nature* **461**, 640-643 (2009).
13. Xu X, You H, Du K, F. H. An *Archaeopteryx*-like theropod from China and the origin of Avialae. *Nature* **475**, 465-470 (2011).
14. Xu X, Zhang FC. A new maniraptoran with long metatarsalian feathers from China. *Naturwissenschaften* **92**, 173-177 (2005).
15. Zhang F, Zhou Z, Xu X, Wang X. A juvenile coelurosaurian theropod from China indicates arboreal habits. *Naturwissenschaften* **89**, 394-398 (2002).
16. Godefroit P, Demuynck H, Dyke G, Hu D, Escuillié F, Claeys P. Reduced plumage and flight ability of a new Jurassic paravian theropod from China. *Nature Communications* **4**, 1394 (2013).
17. Godefroit P, Cau A, Hu DY, Escuillié F, Wu WH, Dyke G. A Jurassic avialan dinosaur from China resolves the early phylogenetic history of birds. *Nature* **498**, 359-362 (2013).
18. Witmer LM. An icon knocked from its perch. *Nature* **475**, 458-459 (2011).
19. Wang XL, Zhou ZH, Zhang FC, Xu X. A nearly completely articulated rhamphorhynchoid pterosaur with exceptionally well-preserved wing membranes and "hairs" from InnerMongolia, northeast China. *Chinese Science Bulletin* **47**, 226-230 (2002).

20. Ji Q, Yuan CX. Discovery of two kinds of protofeathered pterosaurs in the Mesozoic Daohugou Biota in the Ningcheng region and its stratigraphic and biologic significances. *Geological Review (China)* **48**, 221-224 (2002).
21. Zhou ZH, Jin F, Wang Y. Vertebrate assemblages from the Middel-Late Jurassic Yanliao Biota in northeast China. *Earth Science Frontiers* **17**, 252-254 (2010).
22. Liu YQ, Kuang H, Jiang X, Peng N, Xu H, Sun H. Timing of the earliest known feathered dinosaurs and transitional pterosaurs older than the Jehol Biota. *Palaeogeography Palaeoclimatology Palaeoecology* **323-325**, 1-12 (2012).
23. Xu X*, et al.* A new feathered maniraptoran dinosaur fossil that fills a morphological gap in avian origin. *Chinese Science Bulletin* **54**, 430-435 (2009).
24. Agnolín FL, Novas FE. *Avian ancestors-A review of the Phylogenetic relationships of the theropods Unenlagiidae, Microraptoria, Anchiornis and Scansoriopterygidae.* Springer (2013).
25. Turner A, H., Makovicky PJ, Norell MA. A review of dromaeosaurid systematics and paravian phylogeny. *Bulletin of the American Museum of Natural History* **371**, 1-206 (2012).
26. Brusatte SL, Lloyd GT, Wang SC, Norell MA. Gradual assembly of avian body plan culminated in rapid rates of evolution across the dinosaur-bird transition. *Current Biology* **24**, 2386-2392 (2014).
27. Goloboff PA, Farris J, Nixon KC. TNT, a free program for phylogenetic analysis. *Cladistics* **24**, 774-786 (2008).
28. Brusatte S, Lloyd G, Wang S, Norell M. Gradual assembly of avian body plan culminated in rapid rates of evolution across the dinosaur-bird transition. *Current Biology* **24**, 1-7 (2014).
29. Li QG*, et al.* Plumage color patterns of an extinct dinosaur. *Science* **327**, 1369–1372 (2010).
30. Li QG*, et al.* Reconstruction of *Microraptor* and the evolution of iridescent plumage. *Science* **335**, 1215-1219 (2012).
31. Clarke JA*, et al.* Fossil Evidence for Evolution of the Shape and Color of Penguin Feathers. *Science* **330**, 954–957 (2010).
32. Preston LJ, Shuster, J., Fernández-Remolar, D., Banerjee, N. R., Osinski, G. R., & Southam, G. The preservation and degradation of filamentous bacteria and biomolecules within iron oxide deposits at Rio Tinto, Spain. *Geobiology* **9**, 233-249 (2011).
33. Colleary C*, et al.* Chemical, experimental, and morphological evidence for diagenetically altered melanin in exceptionally preserved fossils. *Proceedings of the National Academy of Sciences of the United States of America* **112**, 12592-12597 (2015).
34. McNamara ME, Briggs DEG, Orr PJ, Field DJ, Wang Z. Experimental maturation of feathers: implications for reconstructions of fossil feather colour. *Biology Letters* **9**, 20130184 (2013).
