# Supplementary material for: A bony-crested Jurassic dinosaur with evidence of iridescent plumage highlights complexity in early paravian evolution
Source: Nat Commun. 2018 Jan 15;9:217. doi: 10.1038/s41467-017-02515-y (PMC5768872; doi:10.1038/s41467-017-02515-y)
Supplement: Supplementary file 3 — Description of Additional Supplementary Files [file 41467_2017_2515_MOESM3_ESM.pdf]

File Name: Supplementary Data 1
Description: Matrix derived from Xu et al 2015 (*1*) for phylogenetic analysis, which is saved in Nona format.

File Name: Supplementary Data 2
Description: Matrix derived from Brusatte et al 2014 (*2*) for phylogenetic analysis, which is saved in Nona format.

1. X. Xu *et al.*, A bizarre Jurassic maniraptoran theropod with preserved evidence of membranous wings. *Nature* **521**, 70-73 (2015).
2. S. Brusatte, G. Lloyd, S. Wang, M. Norell, Gradual assembly of avian body plan culminated in rapid rates of evolution across the dinosaur-bird transition. *Current Biology* **24**, 2386-2392 (2014).
